# A Novel α-Calcitonin Gene-Related Peptide Analogue Protects Against End-Organ Damage in Experimental Hypertension, Cardiac Hypertrophy, and Heart Failure

**DOI:** 10.1161/CIRCULATIONAHA.117.028388

**Published:** 2017-07-24

**Authors:** Aisah A. Aubdool, Pratish Thakore, Fulye Argunhan, Sarah-Jane Smillie, Moritz Schnelle, Salil Srivastava, Khadija M. Alawi, Elena Wilde, Jennifer Mitchell, Keith Farrell-Dillon, Daniel A. Richards, Giuseppe Maltese, Richard C. Siow, Manasi Nandi, James E. Clark, Ajay M. Shah, Anette Sams, Susan D. Brain

**Affiliations:** From Cardiovascular Division, BHF Centre of Research Excellence and Centre of Integrative Biomedicine, King’s College London, United Kingdom (A.A.A., F.A., S.-J.S., S.S., K.M.A., E.W., J.M., K.F.-D., G.M., R.C.S., S.D.B.); Institute of Pharmaceutical Sciences, King’s College London, United Kingdom (P.T., M.N.); Cardiovascular Division, BHF Centre of Research Excellence, James Black Centre, King’s College London, United Kingdom (M.S., D.A.R., A.M.S.); Department of Cardiology and Pneumology, Medical Center Goettingen, Germany (M.S.); Cardiovascular Division, BHF Centre of Research Excellence, Rayne Institute, St Thomas’ Hospital, King’s College London, United Kingdom (J.E.C.); Novo Nordisk A/S, Diabetic Complications Biology, Novo Nordisk Park, Maaloev, Denmark (A.S.); and Department of Clinical Experimental Research, Glostrup Research Institute, Rigshospitalet, Denmark (A.S.).

**Keywords:** heart failure, hypertension, inflammation, oxidative stress, receptors, calcitonin gene-related peptide

## Abstract

Research into the therapeutic potential of α-calcitonin gene–related peptide (α-CGRP) has been limited because of its peptide nature and short half-life. Here, we evaluate whether a novel potent and long-lasting (*t*_½_ ≥7 hours) acylated α-CGRP analogue (αAnalogue) could alleviate and reverse cardiovascular disease in 2 distinct murine models of hypertension and heart failure in vivo.

**Methods:**

The ability of the αAnalogue to act selectively via the CGRP pathway was shown in skin by using a CGRP receptor antagonist. The effect of the αAnalogue on angiotensin II–induced hypertension was investigated over 14 days. Blood pressure was measured by radiotelemetry. The ability of the αAnalogue to modulate heart failure was studied in an abdominal aortic constriction model of murine cardiac hypertrophy and heart failure over 5 weeks. Extensive ex vivo analysis was performed via RNA analysis, Western blot, and histology.

**Results:**

The angiotensin II–induced hypertension was attenuated by cotreatment with the αAnalogue (50 nmol·kg^–1^·d^–1^, SC, at a dose selected for lack of long-term hypotensive effects at baseline). The αAnalogue protected against vascular, renal, and cardiac dysfunction, characterized by reduced hypertrophy and biomarkers of fibrosis, remodeling, inflammation, and oxidative stress. In a separate study, the αAnalogue reversed angiotensin II–induced hypertension and associated vascular and cardiac damage. The αAnalogue was effective over 5 weeks in a murine model of cardiac hypertrophy and heart failure. It preserved heart function, assessed by echocardiography, while protecting against adverse cardiac remodeling and apoptosis. Moreover, treatment with the αAnalogue was well tolerated with neither signs of desensitization nor behavioral changes.

**Conclusions:**

These findings, in 2 distinct models, provide the first evidence for the therapeutic potential of a stabilized αAnalogue, by mediating (1) antihypertensive effects, (2) attenuating cardiac remodeling, and (3) increasing angiogenesis and cell survival to protect against and limit damage associated with the progression of cardiovascular diseases. This indicates the therapeutic potential of the CGRP pathway and the possibility that this injectable CGRP analogue may be effective in cardiac disease.

## Abstract 2

Supplemental Digital Content is available in the text.

**Editorial, see p 384**

Calcitonin gene-related peptide (CGRP) is a member of the calcitonin family of peptides. CGRP is primarily localized to sensory nerves, although nonneuronal sources are reported.^1^ The major CGRP receptor is formed by the coexpression of calcitonin receptor-like receptor (CLR) with receptor activity–modifying protein-1 (RAMP1).^1^ These receptors are found throughout the cardiovascular system, specifically in the media, intima, and endothelial layer of blood vessels. Although CGRP is established as a potent vasodilator, there is little evidence that sufficient endogenous CGRP is released to influence physiological blood pressure regulation, although CGRP-containing nerves surround all cardiovascular tissues. Several CGRP receptor antagonists and antibodies developed as migraine therapies have minimal effect on blood pressure in healthy individuals.^1,2^ Evidence that CGRP plays a role in cardiovascular protection arises from acute studies where CGRP has been administered in rodent models of hypertension,^3,4^ using spontaneously hypertensive rats^5,6^ and α-CGRP–specific knockout (KO) mice.^7^ The beneficial effects of the native CGRP peptide have also been observed when administered for up to 24 hours to patients with congestive heart failure with no evidence of tolerance.^8,9^

Although systemic endogenous CGRP levels are raised in some conditions such as pregnancy, it has proven difficult to raise endogenous CGRP levels to provide cardiovascular benefit. There is little evidence that stimulation of the major sensory nerve-localized transient receptor potential (TRP) channels (TRPV1 or TRPA1) releases CGRP to play a primary protective endogenous role in hypertension.^10,11^ This is despite knowledge that activation of TRP channels expressed on sensory nerves induces CGRP-dependent vasodilation in peripheral tissues such as skin.^12^ Of note, TRPA1 activation using nitroxyl^13^ mediates inotropic effects in the failing heart,^14^ but the importance of CGRP is debated.^15^ Some CGRP/calcitonin KO mouse strains possess a raised blood pressure at baseline, indicating a potential role of endogenous CGRP.^1,16^ We have shown that α-CGRP–specific KO mice have normal baseline blood pressure but enhanced hypertension following angiotensin II (AngII) infusion for up to 28 days, in comparison with wild-type mice.^7^ This was associated with aortic hypertrophy and endothelial dysfunction observed as loss of endothelial nitric oxide synthase and evidence of oxidative stress.^7^

This and related knowledge, together with the understanding that global human RAMP1 transgenic mice are protected from hypertension,^17^ led us to hypothesize that a stabilized α-CGRP agonist with the ability to remain active over prolonged periods would elicit cardioprotective properties. This first α-CGRP analogue (αAnalogue) is acylated with an albumin-binding fatty acid moiety that allows reversible albumin binding (patent WO 2011/051312 A1).^18,19^ It has similar pharmacological properties to the native CGRP peptide, but exhibits prolonged action and improved pharmacokinetic properties with a half-life of >7 hours that benefited a model of type 2 diabetes mellitus.^18,19^ The current study demonstrates that the stabilized αAnalogue^18^ protects against the development of AngII-induced hypertension and abdominal aortic constriction (AAC)–induced cardiac hypertrophy and heart failure in mice for several weeks. We have determined mechanisms by which this αAnalogue can reverse vascular, renal, and cardiac damage. To our knowledge, this is the first detailed study using an α-CGRP agonist that has improved stability over the native peptide and illustrates the potential of the CGRP pathway as a therapeutic target and injectable stabilized CGRP agonists as therapeutic agents.

## Methods

A detailed Methods is provided in the online-only Data Supplement.

### Animals

Experiments complied with ARRIVE (Animal Research: Reporting In vivo Experiments) guidelines, in accordance with the UK Home Office Animals (Scientific Procedures) Act, 1986 and approved by the local Animal Care and Ethics Committee. Male mice, CD1 or C57BL/6J (12–18 weeks of age; Charles River) were used.

### AngII Murine Hypertension Model

Mice were infused with AngII (1.1 mg·kg^–1^·d^–1^) or saline (control) continuously for 14 days via osmotic mini pumps, as previously.^7^ Mice were treated daily with αAnalogue (50 nmol·kg^–1^·d^–1^, SC) or vehicle (0.219 mol/L mannitol, 5% hydroxypropyl-β-cyclodextrin, 1.6% ammonium acetate at pH6.5) for 14 days or at 7 days onward for therapeutic dosing.

### Cardiac Hypertrophy Murine Model

Mice were surgically subjected to pressure overload–induced cardiac hypertrophy and heart failure^20^ for 5 weeks and treated daily with αAnalogue (50 nmol·kg^–1^·d^–1^, SC) or vehicle.

### Measurement of Cutaneous Blood Flow

Blood flow was assessed in the ear, leg, or paw using the Full-field Laser Perfusion Imager (Moor Instruments) on anesthetized mice.^12^ To investigate the local effects of αAnalogue, mice were pretreated with the CGRP antagonist BIBN4096 (0.3 mg/kg, IV) or control (neutralized saline) followed by αAnalogue injection (100 pmol daily, ipsilateral ear) or vehicle (contralateral ear). In separate experiments, blood flow in the periphery was measured following systemic treatment of the αAnalogue.

### Measurement of Blood Pressure

Blood pressure, heart rate, and activity were measured using a radiotelemetry (PA-C10, DSI), as previously described^10–12^ in AngII-infused C57BL/6J mice. For the characterization of the systemic dose of *α*Analogue, blood pressure was measured by tail-cuff plethysmography (CODA 8, Kent Scientific) in conscious mice.^7,10^ Following AAC-induced cardiac hypertrophy, blood pressure was measured via carotid artery in anesthetized mice.

### Echocardiography

In vivo cardiac function was assessed using a Vevo 2100 Imaging System with a 40-MHz linear probe (Visualsonics).^20^ Data analysis was performed with Vevo 2100 software v.1.2.1 (Visualsonics).

### Light Aversion Assay

Light aversion (10 minutes, 1000 lux) was determined at baseline and 2 hours following injection of αAnalogue, vehicle, or positive control glyceryl trinitrate (320 nmol/kg, IV). See the online-only Data Supplement.

### Glucose Tolerance Test

Mice were fasted for 6 hours and treated with glucose (1 g/kg, IP). Blood glucose level was determined at baseline and stated time points using a One Touch Vita glucose meter (Lifescan).

### RNA Preparation and Real-Time Quantitative Polymerase Chain Reaction

Total RNA was extracted using the Qiagen RNeasy Microarray Mini Kit (Qiagen), followed by reverse transcription into cDNA (Applied Biosystems, Life technologies Ltd). Quantitative polymerase chain reaction was performed with a SYBR-green–based polymerase chain reaction mix (Sensi-Mix, SYBR-green No ROX; Bioline). Primer details are listed in online-only Data Supplement Table I.

### Western Blotting

Western blot analysis was performed in aorta, mesentery, heart, and kidney as previously described.^7^ Antibody details are listed in the online-only Data Supplement.

### Quantification of Noradrenaline and Cytokines Using Enzyme-Linked Immunosorbent Assay

After 14 days of AngII or saline infusion, plasma and kidney samples were collected for determination of noradrenaline and inflammatory cytokines (interleukin-6, tumor necrosis factor-α) by using standard enzyme-linked immunosorbent assay as described in the online-only Data Supplement.

### Histology

Aorta, heart, and kidney tissues were fixed in 4% paraformaldehyde, as previously described.^7^ Staining protocol and antibody details are listed in the online-only Data Supplement.

### Statistical Analysis

Results are expressed as mean±SEM. Statistical analysis was performed using an unpaired 2-tailed Student *t* test, 1-way or repeated-measures 2-way ANOVA followed by Bonferroni post hoc test. *P*<0.05 was considered to represent a significant difference.

## Results

### Effects of Local Administration of the αAnalogue on Vascular Blood Flow

**Figure 1.**
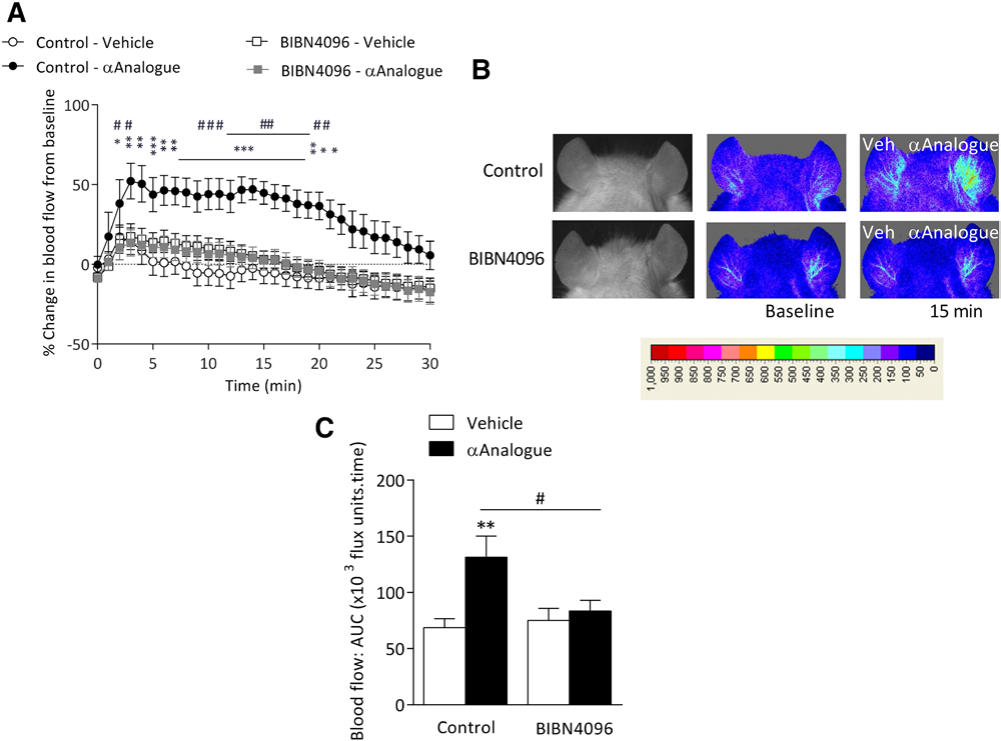
**α-CGRP analogue (αAnalogue) increases vascular blood flow via CGRP receptors.** Blood flow monitored using Full-field Laser Perfusion Imager in the ear vasculature of mice pretreated with control (saline) or CGRP receptor antagonist BIBN4096 (0.3 mg/kg, IV) at baseline and following intradermal injection of αAnalogue (100 pmol, daily) or vehicle (Veh) (15 µL, n=6). **A**, Blood flow responses expressed as % change from baseline. **B**, Representative Full-field Laser Perfusion Imager pictures alongside gray/black photo showing blood flow at baseline and 15 minutes after treatment. **C**, Blood flow assessed by area under the curve (AUC) for 30 minutes following vehicle or αAnalogue administration (n=6). Data showed as mean±SEM. *P<0.05, **P<0.01, ***P<0.001 versus vehicle-treated; #*P*<0.05, ##*P*<0.01, ###*P*<0.001 for αAnalogue treated (**A**, repeated-measures 2-way ANOVA + Bonferroni post hoc test; **C**, 2-way ANOVA + Bonferroni post hoc test). α-CGRP indicates α-calcitonin gene–related peptide.

Initial studies determined the ability of the αAnalogue to increase blood flow via the CGRP receptor (CLR/RAMP1) pathway. Intradermal administration of the αAnalogue increased blood flow in a dose-dependent manner in the skin of anesthetized naive mice as determined by laser perfusion imaging (Figure 1A and 1B, online-only Data Supplement Figure I). This effect was abolished by the selective nonpeptide CGRP receptor antagonist BIBN4096 (Figure 1A through 1C).

### Systemic Treatment With the αAnalogue Protects Against Hypertension

**Figure 2.**
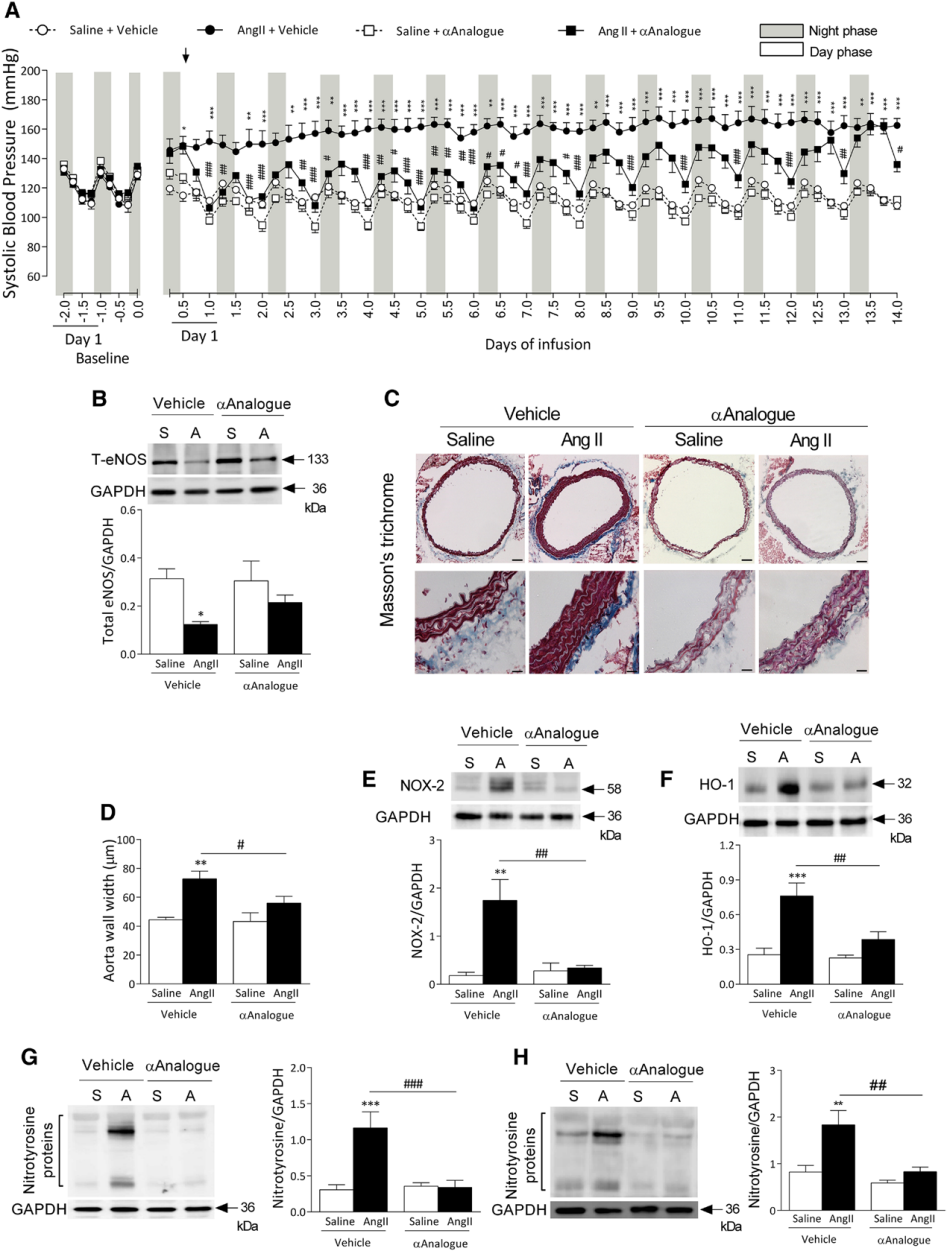
**Daily systemic treatment with α-CGRP analogue (αAnalogue) protects against angiotensin II (AngII)–induced hypertension and vascular damage.** Mice were infused with AngII (**A**, 1.1 mg·kg^–1^·d^-–1^) or control (S, saline) for 14 days and treated daily with vehicle (V) or αAnalogue (50 nmol/kg, SC). **A**, Systolic blood pressure was measured by radiotelemetry. Results expressed as 6-hour average. Mice experience a 12/12 hour light/dark cycle, with the dark cycle shown in the gray striped area. Arrow represents the start of daily treatment. **B**, Protein expression of total eNOS in aorta (n=4–5). **C**, Representative images of Masson trichrome-stained aortic sections. **D**, Quantification of smooth muscle wall width (n=4–5; scale bars, 100 µm). Protein expression of NADPH oxidase-2 (NOX-2) (**E**), heme oxygenase-1 (HO-1) (**F**), nitrotyrosine in aorta (n=4–6) (**G**). **H**, Protein expression of nitrotyrosine in mesenteric vessels (n=6–7). Results shown as mean±SEM. *P<0.05, **P<0.01, ***P<0.001 versus vehicle-treated saline-infused; #*P*<0.05, ##*P*<0.01, ###*P*<0.001 for αAnalogue-treated AngII-infused versus vehicle-treated AngII-infused (**A**, repeated-measures 2-way ANOVA + Bonferroni post hoc test; **B** through **H**, 2-way ANOVA + Bonferroni post hoc test). α-CGRP indicates α-calcitonin gene–related peptide; and eNOS, endothelial nitric oxide synthase.

Systemic injection of the αAnalogue (10–100 nmol/kg, SC) induced a dose-dependent decrease in blood pressure at 1 to 6 hours, with significance observed at 100 nmol/kg in comparison with vehicle treatment, assessed by tail-cuff in naive mice (online-only Data Supplement Figure II). A 50 nmol/kg dose was chosen for further studies, because the hypotensive response had recovered by 24 hours in all mice. It is important to note that the blood pressure effects of the αAnalogue (50 nmol/kg, SC) were not significantly different from vehicle-treated mice (Figure 2A, online-only Data Supplement Figure III). All mice demonstrated normal diurnal variations in cardiovascular hemodynamics at baseline, followed by a hypertensive phenotype post–AngII infusion (Figure 2, online-only Data Supplement Figure III). Daily systemic treatment with the αAnalogue throughout the 14 days markedly blunted AngII-induced hypertension (Figure 2A, online-only Data Supplement Figure III). No significant change in heart rate or activity was observed among treatment groups (online-only Data Supplement Figure III).

In the AngII-infused mice, treatment with the αAnalogue leads to a reproducible reduction in blood pressure, with a similar reduction observed at day 1 to day 14 of treatment (online-only Data Supplement Figure IV), although there was a reduced hypotensive response to the αAnalogue on the last day. However, the protective activity was clearly maintained, with marked reduction in vascular remodeling and oxidative stress (Figure 2). AngII infusion increased water consumption from day 3, consistent with previous findings,^21^ and this was reduced by the αAnalogue treatment throughout the time course (online-only Data Supplement Figure VA and VB). Typically, AngII reduced body weight, which was absent in αAnalogue-treated mice (online-only Data Supplement Figure VC). Food intake was not affected in any treatment groups (online-only Data Supplement Figure VD).

### Systemic Treatment With the αAnalogue Does Not Affect Normal Behavioral Responses or Glucose Homeostasis

One of the limiting factors in the potential use of CGRP agonists therapeutically is that they may cause indications relevant to migraine, flushing, or metabolic changes.^1^ We therefore examined its effects on activity and core body temperature by radiotelemetry, behavioral responses using a light aversion assay, and signs of flushing by assessing peripheral blood flow. Treatment with the αAnalogue (50 nmol/kg, SC) had no effect on activity (online-only Data Supplement Figure IIID) or light avoidance in comparison with baseline (online-only Data Supplement Figure VI), unlike glyceryl trinitrate, an established inducer of migraine symptoms. Neither acute nor chronic systemic treatment of the αAnalogue had a significant effect on skin blood flow (online-only Data Supplement Figure VII). We found no significant change in core body temperature (online-only Data Supplement Figure VIII) or in glucose homeostasis (online-only Data Supplement Figure IX) with the αAnalogue treatment in comparison with vehicle.

### αAnalogue Protects Against AngII-Induced Vascular Changes in the Aorta and Mesentery

AngII infusion caused endothelial dysfunction, revealed by a significant decrease in endothelial nitric oxide synthase expression and vascular hypertrophy, with increased aortic wall thickness. These were not observed in mice treated with the αAnalogue (Figure 2B through 2D). AngII upregulated NADPH oxidase-2 (NOX-2) expression, accompanied by increased vascular aortic oxidative stress, as reflected by an increase in the stress-response protein heme oxygenase-1 (HO-1) and nitration of protein tyrosine residues, an indication of peroxynitrite formation in vehicle-treated AngII-infused mice. These responses were attenuated by the αAnalogue (Figure 2E through 2G). Although we observed no changes in endothelial nitric oxide synthase expression following AngII infusion in mesenteric vessels (online-only Data Supplement Figure X), there was a marked increase in nitrosative stress, which was blocked by the αAnalogue (Figure 2H).

### αAnalogue Treatment Protects Against AngII-Induced Cardiac Remodeling, Fibrosis, and Oxidative Stress

**Figure 3.**
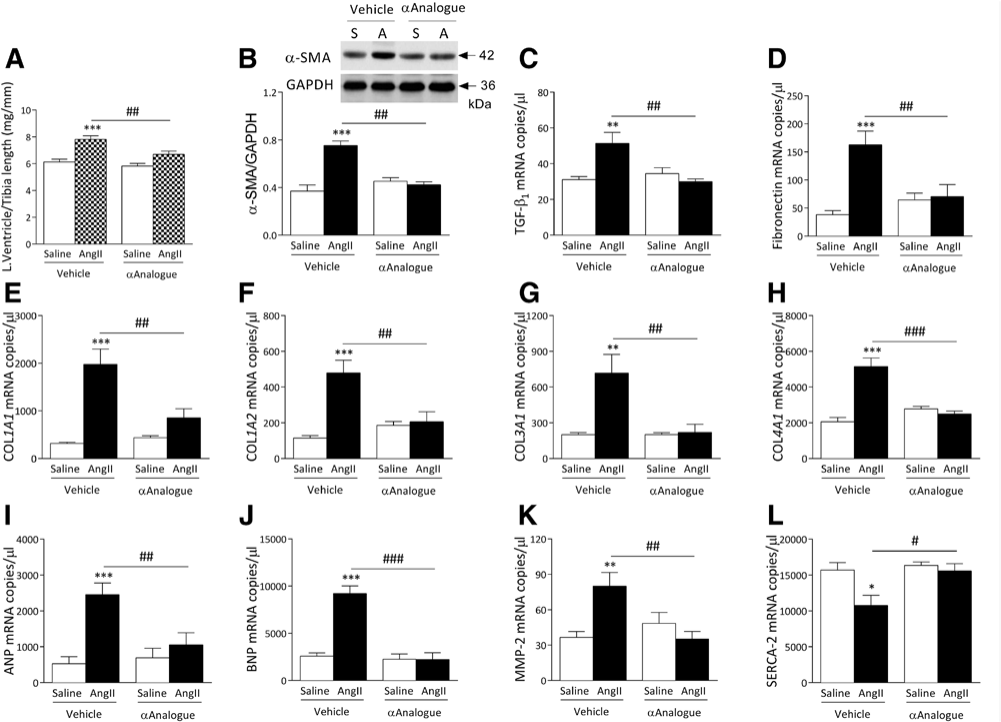
**α-CGRP analogue (αAnalogue) protects against angiotensin II (AngII)–induced cardiac hypertrophy and fibrosis.** Mice were treated as in Figure 2 (AngII, A; Saline, S). **A**, Left ventricle weight normalized to tibia length ratio (mg/mm). **B**, Protein expression of α-smooth muscle actin (α-SMA) in heart (n=5). mRNA expression measured by real-time quantitative polymerase chain reaction for transforming growth factor-β1 (TGF-β_1_) (**C**), fibronectin (**D**), collagen type 1 α1 (COL*1A1*) (**E**), collagen type 1 α2 (COL*1A2*) (**F**), collagen type 3 α1 (COL*3A1*) (**G**), collagen type 4 α1 (COL*4A1*) (**H**), atrial natriuretic peptide (ANP) (**I**), brain natriuretic peptide (BNP) (**J**), matrix metalloproteinase-2 (MMP-2) (**K**), and sarcoplasmic reticulum Ca^2+^ ATPase-2 (SERCA-2) (**L**) in heart (n=5–11). Results expressed as copy numbers per microliter normalized to hypoxanthine-guanine phosphoribosyltransferase, B_2_M and β-actin, and showed as mean±SEM. *P<0.05, **P<0.01, ***P<0.001 versus vehicle-treated saline-infused; #*P*<0.05, ##*P*<0.01, ###*P*<0.001 versus vehicle-treated AngII-infused (2-way ANOVA + Bonferroni post hoc test). α-CGRP indicates α-calcitonin gene–related peptide.

AngII-induced cardiac hypertrophy was reduced by the αAnalogue (Figure 3A, online-only Data Supplement Table II). Hearts of vehicle-treated but not αAnalogue-treated AngII-infused mice showed increased protein expression of α-smooth muscle actin and mRNA expression of several cardiac stress markers, including transforming growth factor-β_1_, a major driver of cardiac fibrosis, extracellular matrix remodeling markers such as connective tissue growth factor, fibronectin, collagen type 1, 3, and 4, and natriuretic peptides such as atrial natriuretic peptide and brain natriuretic peptide (Figure 3B through 3J, online-only Data Supplement Table III). Transforming growth factor-β_1_ induces matrix metalloproteinase-2 in fibroblasts contributing to cardiac remodeling.^22^ Daily treatment with the αAnalogue protected against AngII-induced changes in matrix metalloproteinase-2 and the corresponding tissue inhibitor of metalloproteinase-2 mRNA expression in the hearts (Figure 3K, online-only Data Supplement Table III). The cardiac muscle–specific sarcoplasmic reticulum Ca^2+^ ATPase-2 is important in Ca^2+^ handling, and its expression decreases under pathological conditions, leading to contractile dysfunction.^23^ The αAnalogue protected against AngII-induced decrease in sarcoplasmic reticulum Ca^2+^ ATPase-2 mRNA expression (Figure 3L).

**Figure 4.**
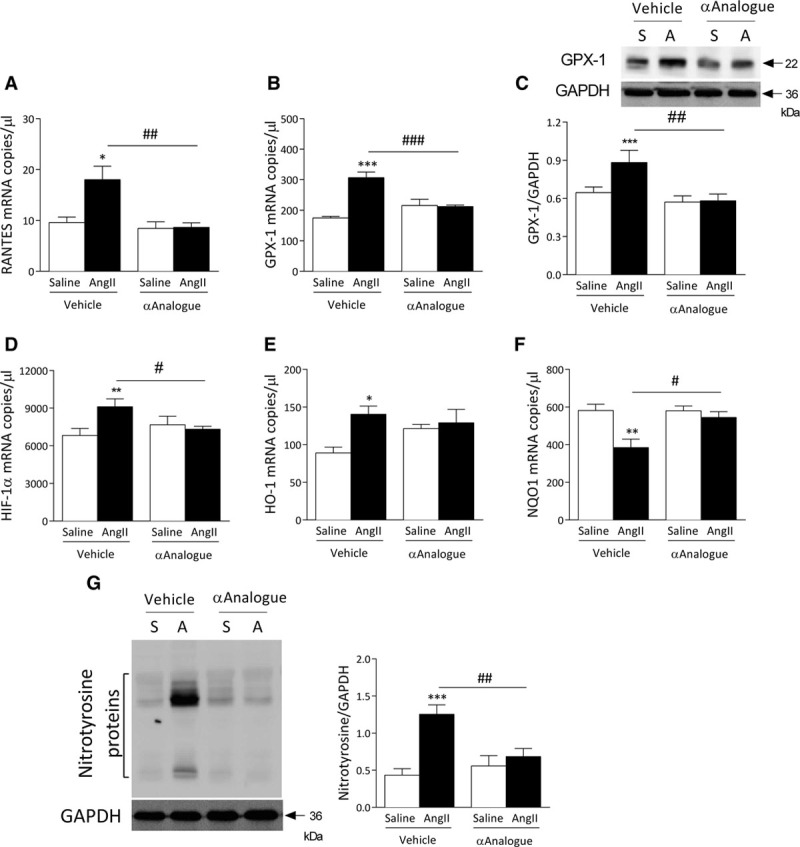
**Daily systemic treatment with α-CGRP analogue (αAnalogue) protects against angiotensin II (AngII)–induced cardiac inflammation and oxidative stress.** Mice were treated as in Figure 2 (AngII, A; Saline, S). mRNA expression measured by real-time quantitative polymerase chain reaction (n=5–11) for RANTES (**A**) and glutathione peroxidase-1 (GPX-1) (n=6–11) (**B**). **C**, Protein expression of GPX-1 in heart (n=5). mRNA expression for hypoxia-inducible factor-1 (HIF-1α) (**D**), heme oxygenase-1 (HO-1) (**E**), and NADPH dehydrogenase quinone-1 (NQO1) (n=5–11) (**F**). Results expressed as copy numbers per microliter normalized to hypoxanthine-guanine phosphoribosyltransferase, B_2_M and β-actin, and showed as mean±SEM. **G**, Protein expression of nitrotyrosine (n=5) in heart (n=6–7). *P<0.05, **P<0.01, ***P<0.001 versus vehicle-treated saline-infused; #*P*<0.05, ##*P*<0.01, ###*P*<0.001 versus vehicle-treated AngII-infused (2-way ANOVA + Bonferroni post hoc test). α-CGRP indicates α-calcitonin gene–related peptide.

The αAnalogue protected against AngII-induced inflammation, as shown by reduced mRNA expression of the nuclear factor kappa B cells and the chemokine RANTES (Figure 4A, online-only Data Supplement Table III). Treatment with the αAnalogue caused no change in the apoptosis regulator B-cell lymphoma-2 (Bcl-2) but resulted in a significant reduction in apoptotic markers such as p53 in AngII-infused hearts (online-only Data Supplement Table III). We next determined the effects of the αAnalogue on oxidative stress. AngII-induced increase in the antioxidant enzyme glutathione peroxidase-1 expression was absent in αAnalogue-treated groups (Figure 4B and 4C). An increase in hypoxia-inducible factor 1α was attenuated by αAnalogue treatment (Figure 4D). AngII-induced changes in the mRNA expression of HO-1 and the phase II detoxifying enzymes NADPH dehydrogenase quinone-1 were absent by daily treatment with the αAnalogue (Figure 4E and 4F). Cardiac nitrosative stress was absent by the αAnalogue treatment in AngII-infused mice (Figure 4G). Collectively, these results show a marked protective effect on the proinflammatory and oxidative pathways associated with AngII-induced cardiovascular disease.

### αAnalogue Protects Against AngII-Induced Renal Fibrosis and Injury

**Figure 5.**
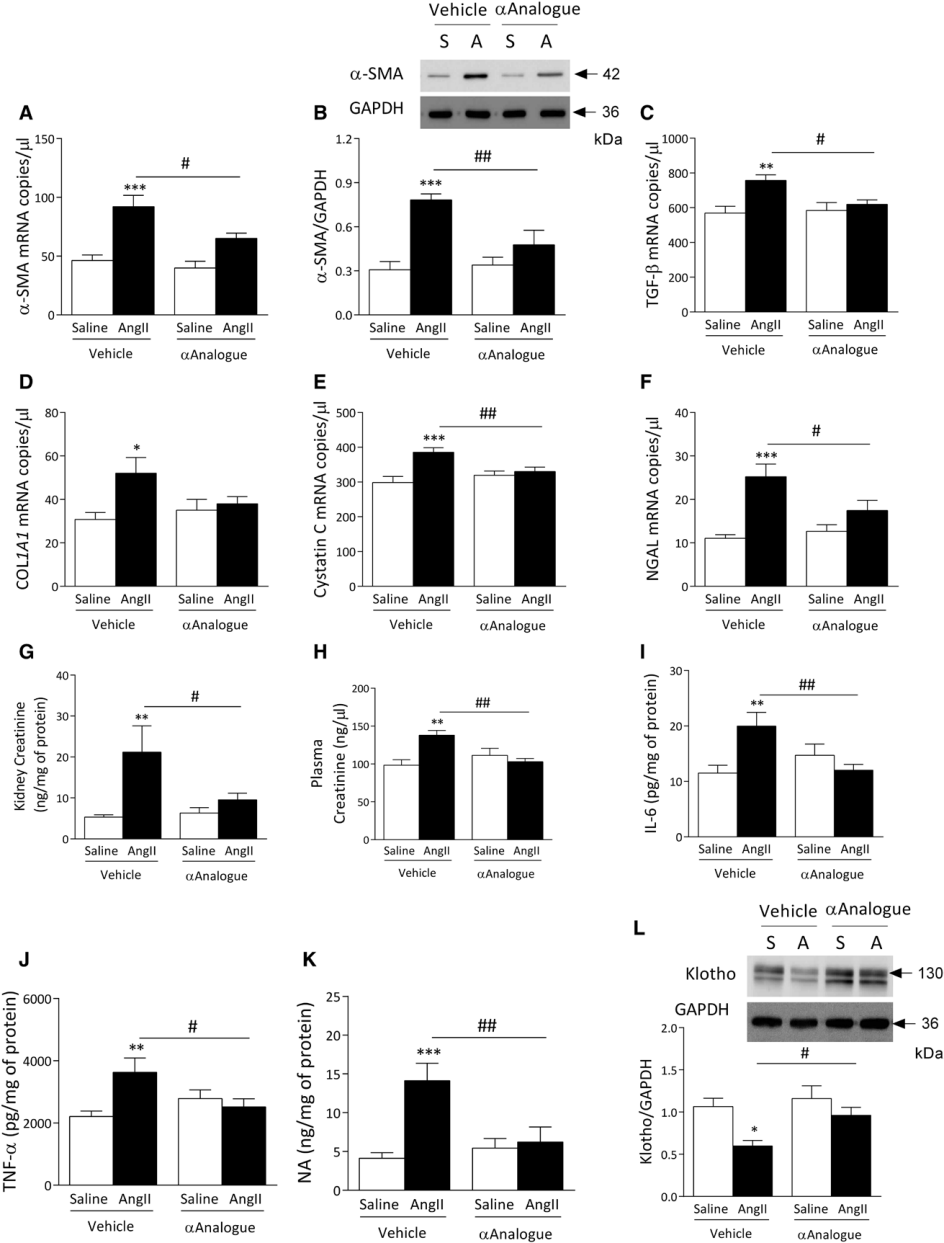
**Daily systemic treatment with α-CGRP analogue (αAnalogue) protects against angiotensin II (AngII)–induced renal fibrosis, dysfunction, and inflammation.** Mice were treated as in Figure 2 (AngII, A; Saline, S). mRNA (**A**) and protein expression of α-SMA (**B**) in kidney. mRNA expression of TGF-β_1_ (**C**), collagen type 1a1 (COL*1A1*) (**D**), cystatin C (**E**), and neutrophil gelatinase-associated lipocalin (NGAL) (**F**) in kidney. Creatinine levels in kidney (**G**) and plasma (**H**) (n=6–8). IL-6 (**I**), TNF-α (**J**), and noradrenaline (NA) (**K**) levels in kidney (n=6–9). **L**, Protein expression of klotho in kidney. mRNA expression measured by real-time quantitative polymerase chain reaction (n=6–8), expressed as copy numbers per microliter normalized to hypoxanthine-guanine phosphoribosyltransferase, B_2_M, and β-actin. Results shown as mean±SEM. *P<0.05, **P<0.01, ***P<0.001 versus vehicle-treated saline-infused; #*P*<0.05, ##*P*<0.01 versus vehicle-treated AngII-infused (2-way ANOVA + Bonferroni post hoc test). α-CGRP indicates α-calcitonin gene–related peptide; IL-6, interleukin 6; α-SMA; α-smooth muscle actin; TGF-β1; transforming growth factor-β1; and TNF-α, tumor necrosis factor-α.

AngII infusion caused renal fibrosis, indicated by upregulation of α-smooth muscle actin expression, increased transforming growth factor-β_1_ and collagen mRNA expression, which were absent in mice cotreated with the αAnalogue (Figure 5A through 5D). The kidneys of AngII-infused mice exhibited renal damage, with increased mRNA expression of cystatin C and neutrophil gelatinase-associated lipocalin, plasma and renal creatinine levels, and mesangial matrix expansion in the glomeruli, which were all absent in mice treated with the αAnalogue (Figure 5E through 5H, online-only Data Supplement Figure XI). AngII infusion resulted in renal inflammation, highlighted by increased interleukin-6 and tumor necrosis factor-α concentrations, which were abolished by the αAnalogue (Figure 5I and 5J). There was a significant increase in localized sympathetic nerve activity, shown by increased noradrenaline content in AngII-infused kidney but not plasma, which was blocked by αAnalogue treatment (Figure 5K, online-only Data Supplemental Figure XII). The decreased noradrenaline levels in the kidney provides evidence that CGRP was able to attenuate the sympathetic activity. Downregulation of renal klotho expression can aggravate AngII-induced renal damage.^24^ Here, the αAnalogue protects against this downregulation (Figure 5L).

### αAnalogue Protects Against AngII-Induced Upregulation of CGRP Receptor RAMP1

**Figure 6.**
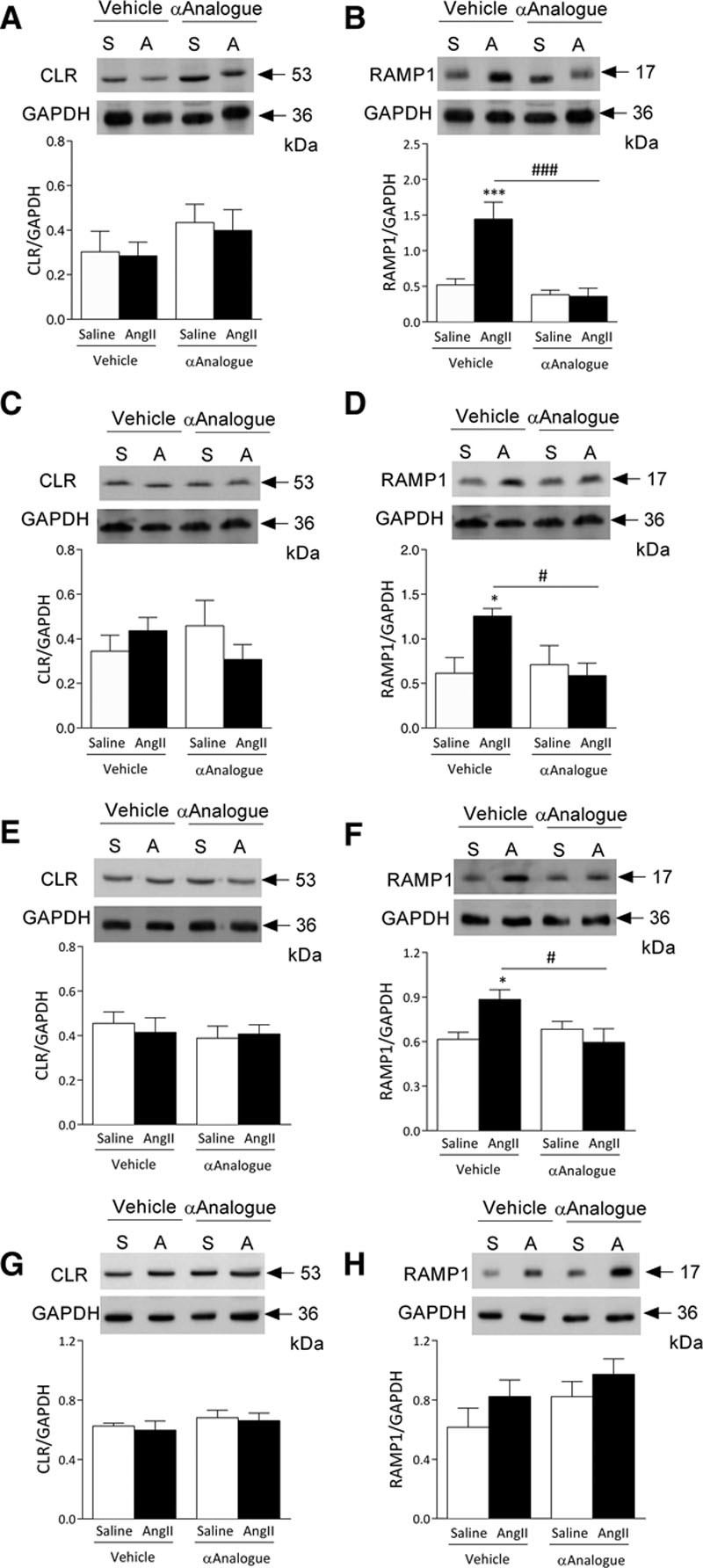
**Effects of daily systemic treatment with α-CGRP analogue (αAnalogue) on CGRP receptor expression in angiotensin II (AngII)–induced hypertension.** Mice were treated as in Figure 2 (AngII, A; saline, S, V, vehicle; α-CGRP analogue; C). Protein expression of calcitonin receptor-like receptor (CLR) (**A**) and receptor-associated membrane protein-1 (RAMP1) (**B**) in aorta (n=6). Protein expression of CLR (**C**) and RAMP1 (**D**) in mesenteric vessels (n=6). Protein expression of CLR (**E**) and RAMP1 (**F**) in heart (n=6–7). Protein expression of CLR (**G**) and RAMP1 (**H**) in kidney (n=5–6). Results showed as mean±SEM.*P<0.05, ***P<0.001 versus vehicle-treated saline-infused; #*P*<0.05, ###*P*<0.001 versus vehicle-treated AngII-infused (2-way ANOVA + Bonferroni post hoc test). α-CGRP indicates α-calcitonin gene–related peptide.

RAMP1 determines the CGRP receptor phenotype when associated with CLR, and CGRP receptors are upregulated in cardiovascular disease models.^1^ We observed a significant increase in CGRP receptor RAMP1 protein expression in the aorta, mesentery, and heart of vehicle-treated AngII-infused mice but not the αAnalogue (Figure 6). There was no significant change in CLR expression observed in different tissues, regardless of treatment (Figure 6). We found no change in endothelin-1 expression in heart or aorta treated with vehicle or the αAnalogue (online-only Data Supplement Figure XIII).

### αAnalogue Limits AngII-Induced Hypertension and Associated Cardiac Remodeling

**Figure 7.**
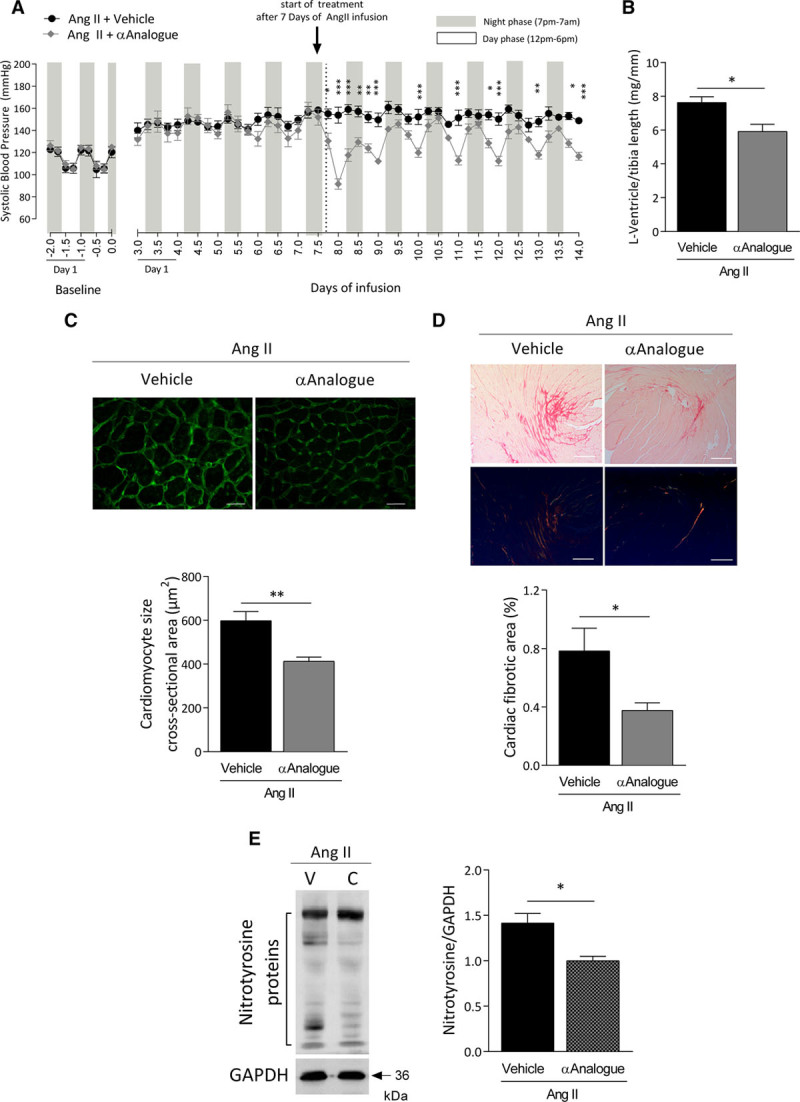
**α-CGRP analogue (αAnalogue) limits angiotensin II (AngII)–induced hypertension**. Mice were infused with AngII (1.1 mg·kg^–1^·d^–1^ for 14 days) and treated daily with vehicle or αAnalogue (50 nmol/kg, SC) at day 7 to 14 of infusion (n=4). **A**, Systolic blood pressure was measured by radiotelemetry. Results expressed as 6-hour average. Mice experience a 12/12 hour light/dark cycle, with the dark cycle shown in the gray striped area. Arrow represents the start of daily treatment. **B**, Left ventricle weight normalized to tibia length ratio (mg/mm). Representative heart sections (**Top**) and analysis (**Bottom**) showing cardiac hypertrophy by cardiomyocyte borders outlined using wheat germ agglutinin (scale bars, 20 µm) (**C**) and cardiac fibrosis by Picrosirius Red staining (scale bars, 200 µm) (**D**). **E**, Protein expression of nitrotyrosine in heart. Results showed as mean±SEM.*P<0.05, **P<0.01, ***P<0.001 versus vehicle-treated (**A**, repeated-measures 2-way ANOVA + Bonferroni post hoc test; **B** through **E**, 2-way ANOVA + Bonferroni post hoc test). α-CGRP indicates α-calcitonin gene–related peptide.

To determine whether the αAnalogue influences established hypertension, we assessed its effects by starting treatment at day 7 of AngII infusion. Treatment with the αAnalogue attenuated AngII-induced hypertension (Figure 7A, online-only Data Supplement Figure XIV). We observed a significant decrease in Akt and osteopontin, remodeling, and fibrotic markers (transforming growth factor-β_1_, connective tissue growth factor, α-smooth muscle actin, collagen type 1 and 3), NOX-2, and markers of oxidative stress (HO-1, NADPH dehydrogenase quinone-1) in aorta (online-only Data Supplement Table IV). Accordingly, a substantial decrease in cardiac hypertrophy was found, with a significant reduction in left ventricular weight:tibia length ratio (Figure 7B, online-only Data Supplement Table V) and in cardiac fibrosis, remodeling, and inflammation, shown by a significant reduction in collagen type 3, atrial natriuretic peptide, and NOX-2 mRNA expression (online-only Data Supplement Table VI). It is notable that the αAnalogue reduced AngII-mediated increase in cardiomyocyte size and collagen deposition, revealed by wheat germ agglutinin and Picrosirius Red staining, respectively (Figure 7C and 7D). Cardiac nitrosative stress was reversed by the αAnalogue in hypertensive mice (Figure 7E).

### αAnalogue Protects Against AAC-Induced Cardiac Hypertrophy and Heart Failure

**Figure 8.**
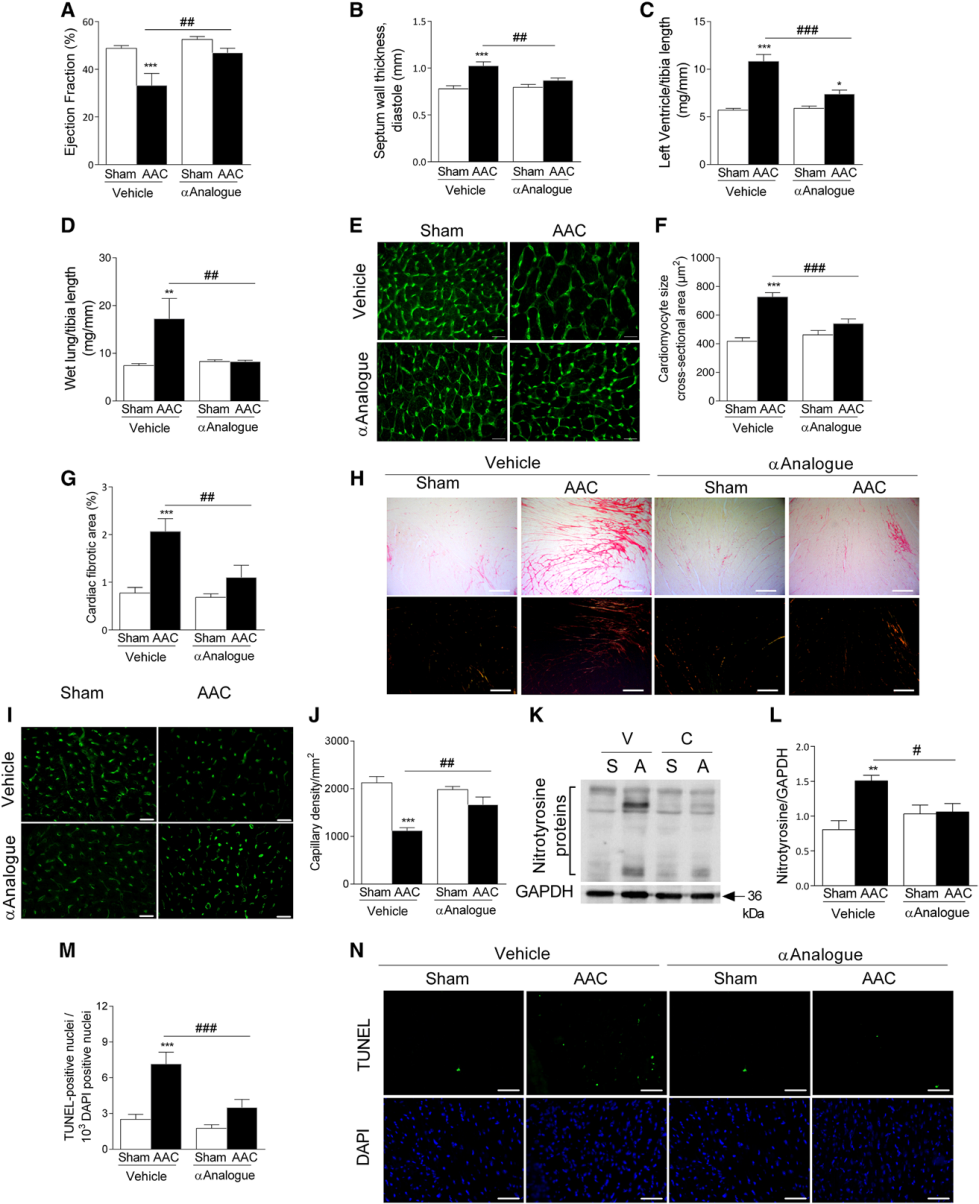
**α-CGRP analogue (αAnalogue) preserves heart function post–AAC-induced cardiac hypertrophy and heart failure.** Mice were treated daily with vehicle and αAnalogue (50 nmol/kg, SC) postsurgery for 5 weeks (n=6–8). Ejection fraction (%) (**A**), septum wall thickness at diastole (mm) (**B**), left ventricle mass normalized to tibia length (mg/mm) (**C**), and wet lung mass (mg) normalized to tibia length (**D**). Representative images (**E**) and quantification of cardiomyocyte cross-sectional area (**F**) in heart using wheat germ agglutinin staining (scale bars, 20 µm). Quantification (**G**) and representative images of fibrosis in heart (**H**) using picrosirius red staining (scale bars, 200 µm). Representative images (**I**) and quantification of capillary density in heart (**J**) using isolectin-B_4_ staining (scale bars, 20 µm). **K** and **L**, Protein expression of nitrotyrosine in heart. Quantification (**M**) and representative images of apoptosis (**N**) using TUNEL staining (scale bars, 50 µm). Results showed as mean±SEM. *P<0.05, **P<0.01, ***P<0.001 versus vehicle-treated sham mice; #*P*<0.05, ##*P*<0.01, ###*P*<0.001 versus vehicle-treated AAC mice (2-way ANOVA + Bonferroni post hoc test). AAC indicates abdominal aorta constriction; α-CGRP, α-calcitonin gene–related peptide; and TUNEL, terminal deoxynucleotidyl transferase dUTP nick-end labeling.

To investigate the cardioprotective effect of CGRP in heart failure, mice were subjected to sham or AAC-induced cardiac hypertrophy and heart failure for 5 weeks. Quantification of in vivo cardiac function revealed that ejection fraction was better preserved in αAnalogue-treated AAC mice, with a reduction in septum wall thickness as shown by echocardiography (Figure 8A and 8B, online-only Data Supplement Figure XV, and online-only Data Supplement Table VII). Chronic treatment with the αAnalogue for 5 weeks was well tolerated, with no significant change in body weight, food and water intake, light avoidance, or blood pressure (online-only Data Supplement Figures XVI and XVII). αAnalogue-treated mice consistently developed less cardiac hypertrophy and fibrosis after AAC than vehicle-treated groups (Figure 8C, 8E through 8H, online-only Data Supplement Table VIII, and online-only Data Supplement Figure XVIII). Insufficient angiogenesis is known as a driver of heart failure,^20^ and left ventricular heart sections subjected to AAC showed reduced capillary density that was reversed by αAnalogue treatment (Figure 8I and 8J). Quantitative immunoblotting showed that phosphorylated p38 mitogen-activated protein kinase levels were significantly increased in αAnalogue-treated AAC hearts (online-only Data Supplement Figure XIX). Mice subjected to AAC showed an increase in cardiac inflammation, oxidative, nitrosative stress, and apoptosis in vehicle-treated but not αAnalogue-treated groups (Figure 8K through 8N, online-only Data Supplement Figure XVIII).

## Discussion

CGRP is one of the most potent microvascular dilators known, and its protective properties are well established in vitro. However, it has been difficult to harness this information in drug discovery projects involving the cardiovascular system to date, primarily because of the instability of the peptide. This study presents novel mechanistic evidence that chronic systemic treatment with an injected stabilized α-CGRP agonist is effective in the onset and ongoing AngII-induced hypertension and AAC-induced cardiac hypertrophy and heart failure in vivo. The αAnalogue, which possesses a considerably longer half-life than the native CGRP peptide (>7 hours in comparison with <30 minutes) is well tolerated. Specifically, the agonist did not lower systemic blood pressure in naive mouse, but exerted beneficial protective effects at the vascular, renal, and cardiac levels by alleviating fibrosis, remodeling, inflammation, oxidative stress, apoptosis, and preserving overall function in 2 distinct cardiovascular disease models. Our results are consistent with the concept that the CGRP receptor can be an influential regulatory component in cardiovascular disease and that CGRP agonists have potential therapeutic benefits.

The αAnalogue acted as a selective CGRP agonist via the CGRP receptor to mediate vasodilatation. Because the αAnalogue is not orally active, a systemic injectable dose that elicited an initial decrease in blood pressure with full recovery by 24 hours was chosen. This allowed baseline blood pressure to be maintained over the 2- to 5-week protocols in the control/sham mice. Although systemic CGRP infusion previously led to flushing in humans,^25^ we found no undesirable side effects at the selected dose.

Although α-CGRP–specific KO mice have normal basal cardiovascular hemodynamics, they exhibit an enhanced hypertensive phenotype in AngII-induced hypertension.^7^ Indeed, acute injection of the native CGRP peptide was beneficial in hypertensive rats^26^ but effects were short lasting (<10 minutes) because of its short half-life.^27^ In contrast, CGRP infusion for 6 days had beneficial effects in hypertensive rats.^3^ To build on this and elucidate the mechanisms, we show that the αAnalogue protects against AngII-induced increase in blood pressure for 2 weeks. We tested for potential desensitization by the daily administration of the αAnalogue in naive mice and found a reproducible hypotensive effect, with no signs of downregulation of the CGRP pathway in the vasculature. However, there was a reduced effect on hypertension at the 14-day time point, leading us to investigate for other markers of disease. AngII-induced hypertension is associated with weight loss and increased water intake, in addition to hypertension. These were reversed by the αAnalogue throughout. Of interest, blocking the CGRP pathway with antagonists/antibodies against CGRP and its receptor (currently in clinical trials for prevention of migraine^2^) may interfere with this protective pathway and thereby increase cardiovascular risk. However, in the limited clinical studies performed to date, no evidence has been found. It is possible that endogenous levels of CGRP do not reach sufficient levels in humans to mediate protective effects. To our knowledge, this is the first in-depth study in cardiovascular models with a long-acting CGRP agonist where there has been a sustained benefit in protective cardiovascular effects.

AngII-induced vascular dysfunction and remodeling were reversed by the αAnalogue, in keeping with reports that CGRP inhibits smooth muscle cell proliferation by increasing cAMP or inhibiting the ERK1/2 signaling cascade.^28^ CGRP has direct anti-inflammatory and antioxidant effects in endothelial progenitor cells.^29^ Accordingly, the αAnalogue reduced the AngII-induced increase in the endogenous source of ROS^30^ NOX-2, oxidative stress-associated proteins, and nitrosative stress in hypertensive aorta, which was consistent with less nitrosative stress in the hypertensive mesenteric vessels. There was no change in vascular endothelial dysfunction in resistance vessels, complementing our previous findings.^7^

CGRP receptors are upregulated in cardiovascular disease,^1^ with evidence of a pressure-dependent regulation.^12^ This may amplify responses to CGRP, especially in hypertension.^7,17^ Accordingly, RAMP1 expression was increased in hypertensive resistance and conduit vessels. The αAnalogue reduced this effect such that RAMP1 returned to normal expression levels as the cardiovascular system benefited, irrespective of the continuing presence of AngII. This finding suggests that CGRP may act in an autoregulatory manner depending on CGRP peptide/agonist availability.

CGRP has positive chronotropic effects,^31^ and α-CGRP KO mice exhibit exacerbated cardiac dysfunction in pressure overload–induced hypertrophy^32^ and deoxycorticosterone acetate salt–induced hypertension.^33,34^ Here, the αAnalogue had no effect on in vivo heart function, as observed by radiotelemetry or echocardiography in sham mice, but protected against cardiac hypertrophy in AngII-induced hypertension. The αAnalogue downregulated markers of fibrosis, remodeling, and hypertrophy, and protected against the reduction in the cardiac contractile dysfunction marker sarcoplasmic reticulum Ca^2+^ ATPase-2 in hypertensive hearts, as well. Our results correlate with previous in vitro findings^35–37^ and provide in vivo evidence that the αAnalogue has influential antiremodeling effects in the stressed heart, while not directly modulating normal heart activity in control mice.

The αAnalogue reduces cardiac inflammation, as shown by reducing AngII-induced increase in the chemokine RANTES. AngII increases hypoxia-inducible factor 1α mRNA expression, indicating the involvement of fibrotic and oxidative stress pathways, and this was reduced by the αAnalogue. CGRP regulates oxidative stress by the PI3K/Akt and mitogen-activated protein kinase signaling pathways,^38^ and deletion of the CGRP gene exacerbates cardiac oxidative stress in ischemia-reperfusion injury.^39^ The αAnalogue protects against AngII-induced changes in the endogenous antioxidant defense responses and nitrosative stress. A link between CGRP and HO-1 is known.^40^ Activation of HO-1 and NADPH dehydrogenase quinone-1 can counteract hypertension, potentially through generating the vasodilator carbon monoxide^41,42^ and improved endothelial nitric oxide synthase coupling.^43^ Our data build on earlier findings in cardiomyocytes, smooth muscle cells, and α-CGRP KO mice, implying that the αAnalogue acts directly via its receptors on the heart and reduces stress-activated kinases induced by oxidative stress,^44^ adverse tissue remodeling to preserve cardiac function.

Hypertension is associated with renal fibrosis and inflammation that were reduced by the αAnalogue, complementing knowledge that CGRP promotes renal protection in hypertension independently of its vasodilator action.^33,45^ The αAnalogue preserved renal function by reducing mesangial matrix expansion, plasma and kidney creatinine levels, and expression of cystatin C and neutrophil gelatinase-associated lipocalin, the early biomarker of acute kidney injury. AngII increases renal adrenergic receptors^46^ and we found a significant increase in renal noradrenaline content, which was reversed by the αAnalogue. Hence, the αAnalogue may preserve renal function by reducing sympathetic activity. Klotho has nephroprotective effects, and AngII-induced downregulation of klotho aggravates renal damage in hypertension.^24^ This downregulation was rescued by the αAnalogue, and our results further confirm the link between klotho and CGRP.^29^ The upregulation of klotho associated with the use of αAnalogue may translate into enhanced renal protection in hypertension. The primary functional source of CGRP was reported to be independent of the kidney, because chemical denervation of renal sensory afferents removed tissue CGRP but without any beneficial effect in hypertensive mice.^46^ However, our study highlights that mimicking sensory nerve efferent function by using an αCGRP analogue protects against end-organ damage in hypertension, especially renal fibrosis, a strong predictor of clinical progression of kidney disease.

Strikingly, the αAnalogue limits AngII-induced cardiovascular pathologies, ameliorating cardiac remodeling and fibrosis. This highlights the therapeutic potential of the αAnalogue when given in established hypertension. Although the αAnalogue was unable to reduce vascular endothelial dysfunction, it reduced markers of damage such as osteopontin, the downstream regulator of Akt activity,^47^ remodeling, fibrosis, and oxidative stress.^7^ The αAnalogue reduced both vascular and cardiac NOX-2 expression. It is possible that many of the beneficial effects of the αAnalogue in both early or late onset of hypertension are primarily related to a decrease in blood pressure and the resultant reduction in pressure-induced damage in the vasculature, kidney, and heart. Although it is presently difficult to separate the direct effects of the αAnalogue on pressure and cardiac function, it is evident that CGRP has antihypertensive effects and induces protection in a comprehensive manner.

To investigate the cardioprotective effects of the αAnalogue, we subjected mice to AAC and examined cardiac function. The adverse structural remodeling and hypertrophy at 5 weeks post-AAC, with significant reduction in ejection fraction and increasing heart mass with fibrosis, were markedly attenuated in αAnalogue-treated mice, under conditions where blood pressure was similar to αAnalogue and vehicle-treated sham mice. This is consistent with findings where α-CGRP/calcitonin KO mice showed adverse cardiac dysfunction with increased mortality following transverse aortic constriction.^32^ Although CGRP infusion for 24 hours previously improved cardiac performance in patients with chronic congestive heart failure,^8,9^ this is the first demonstration that chronic treatment with a long-lasting CGRP agonist is beneficial and well tolerated. Myocyte apoptosis is well documented in heart failure and CGRP regulates cell survival signaling and antiapoptotic pathway via CLR/RAMP1 in cardiomyocytes.^38,48^ Although the αAnalogue reduces nuclear factor kappa B cells and apoptotic marker p53 expression in our acute AngII model, here, the αAnalogue reduced apoptosis, as revealed in terminal deoxynucleotidyl transferase dUTP nick-end labeling staining with increased p38 mitogen-activated protein kinase phosphorylation in the hypertrophic heart. Similarly, the αAnalogue maintained its antioxidant effects. Development of cardiac hypertrophy is associated with increased cardiomyocyte size and reduced capillary density, which ultimately lead to hypoxia and cell death.^20^ These changes were markedly reduced by the αAnalogue. This finding supports earlier studies where CGRP acted as a proangiogenic factor^32,49^ and suggests that CGRP promotes normal cardiac microvessel development, perhaps via angiogenesis in hypertrophic hearts. Further gene expression studies on ventricle tissues demonstrate that markers of heart failure were abrogated by the αAnalogue.

To our knowledge, this is the first study demonstrating that a long-lasting and stable α-CGRP agonist has the potential to act as a novel therapeutic agent, targeting key mechanisms to benefit cardiovascular dysfunction, with limited adverse pathological changes and side effects. The results support the concept provided by in vitro studies that CGRP protects against adverse remodeling, inflammation, oxidative stress, apoptosis, and end-organ damage in cardiovascular disease, in addition to its vasodilator activity. We thus propose that the CGRP pathway is a therapeutic target for the clinical treatment of cardiovascular disease and, more specifically, that injectable CGRP agonists may benefit heart failure.

## Sources of Funding

This work was supported by British Heart Foundation (Grant#-PG/12/34/29557 and RE/13/2/30182). αAnalogue was supplied by Novo Nordisk. Dr Schnelle was supported by a Deutsche Forschungsgemeinschaft Joint PhD Studentship (IRTG1816). F.A. is an MRC PhD student. E.W. is an MRC NC3Rs PhD student.

## Disclosures

None.

## Supplementary Material

## Clinical Perspective

### What Is New?

- We have used a novel injectable stabilized α-calcitonin gene–related peptide (CGRP) agonist, the first to our knowledge, to analyze the effect on cardiovascular disease in 2 distinct murine models.
- We show that the CGRP agonist is well tolerated and selective in the vasculature.
- The CGRP agonist prevented the onset of and limited angiotensin II–induced hypertension.
- The CGRP agonist also protected against heart failure in the abdominal aortic constriction model, suggesting that this α-CGRP analogue is beneficial in cardiovascular disease.

### What Are the Clinical Implications?

- These preclinical data show that activating the protective CGRP pathway, using selective stabilized agonists, constitutes a novel therapeutic application.
- The results are a stimulus to further develop agonists, including those that are orally active.
- The results highlight the potential development of this injectable stabilized CGRP agonist (α-CGRP analogue) for the treatment of cardiac dysfunction (eg, in advanced heart failure).
