# Supplementary material for: A Novel α-Calcitonin Gene-Related Peptide Analogue Protects Against End-Organ Damage in Experimental Hypertension, Cardiac Hypertrophy, and Heart Failure
Source: Circulation. 2017 Jul 24;136(4):367–83. doi: 10.1161/CIRCULATIONAHA.117.028388 (PMC5519346; doi:10.1161/CIRCULATIONAHA.117.028388)
Supplement: Supplementary file 1 [file cir-136-367-s001.pdf]

SUPPLEMENTAL MATERIAL

Supplemental Methods

Animals

Male mice, CD1 (#022) or C57BL/6J (#027) (12-18 weeks of age), purchased from Charles River (Kent, UK) were used in all experiments. Blood flow experiments were performed on CD1 mice, whilst all other experiments with C57BL/6J mice. Mice were housed in a climatically controlled environment, on a 12-h light/dark cycle, with free access to water and standard food *ad libitum*. All experiments were conducted in accordance with the UK Home Office Animals (Scientific Procedures) Act, 1986 and were approved by the King's College London Animal Care and Ethics Committee. All experiments were conducted in a blinded manner. Animals were randomly assigned to control or treatment groups and the experimenter was blinded towards treatment at the time of experiment.

Cutaneous blood flow measurement by Full-field Laser Perfusion Imager

Cutaneous blood flow was assessed in the whole area of ear, leg or paw area using the Full-field Laser Perfusion Imager (FLPI, Moor Instruments) on anaesthetised mice in their ventral positioned placed on a heating mat, maintained at 36°C, as previously described.[1-3]

Local effects of α-CGRP analogue (αAnalogue, Figure 1, Supplemental Figure 1): CD1 mice were anaesthetised intraperitoneally (*i.p.*) with ketamine (75 mg/kg) and medetomidine (1 mg/kg).[1] Following recording of baseline blood flow in both ears to ensure the haemodynamic vascular responses have stabilised after anaesthesia, mice were injected intravenously (*i.v.*) with vehicle (neutralised saline) or CGRP receptor antagonist (BIBN4096, 0.3mg/kg)[1, 4, 5] and blood flow was resumed for another 5 min. The ipsilateral ear was then injected intradermally (*i.d.*) with αAnalogue[6] (100pmol in 15μl, Novo Nordisk), whilst the contralateral ear received vehicle (0.219M Mannitol, 5% HPCD, 1.6% ammonium acetate at pH6.5). Initial experiments investigated other doses of αAnalogue (1-100pmol in 15μl). Changes in blood flow after these treatments were followed for 30 min. Results are expressed as (1) a measure of maximum % increase in blood flow from baseline or (2) arbitrary flux units ($\times 10^3$ flux units) measured as area under the recorded flux (response curve) versus time for the entire recording period for 30 min following *i.d.* injection.

Systemic effects of αAnalogue (Supplemental Figure 7): C57BL/6J mice were anaesthetised with isoflurane (2%, Abbott Laboratories) in 2l/min O_2 and blood flow in the ear was measured for 5 min. Mice were allowed to recover and injected subcutaneously (*s.c.*) with vehicle (0.219M Mannitol, 5% HPCD, 1.6% ammonium acetate at pH 6.5) or αAnalogue (50nmol/kg, *s.c.*, Novo Nordisk). At 1h following treatment, mice were anaesthetised and blood flow was measured for a further 5 min.

In other experiments, blood flow was measured for 5 min in the paw, leg and ear of mice treated daily with vehicle or αAnalogue (50nmol/kg, *s.c.*, Novo Nordisk) for 14 days. Results are expressed as an average blood flow (flux units) for the 5 min for each area.

Measurement of blood pressure via tail-cuff plethysmography

Blood pressure was measured by tail-cuff plethysmography, using the CODA 8 non-invasive blood pressure acquisition technique system for mice (Kent Scientific), as previously described.[7, 8] All measurements were taken at a thermoneutral ambient room temperature (25-27°C). C57BL/6J mice were warmed on a heating pad for 10 min prior to and during blood pressure recordings. Mice were trained for at least 10 consecutive days prior to baseline blood pressure measurements, to reduce stress-induced changes caused by restraint.

Dose-response characterisation of αAnalogue: Measurements were taken at baseline and 1, 6, 24 and 30h following administration of vehicle (0.219M Mannitol, 5% HPCD, 1.6% ammonium acetate at pH 6.5) or αAnalogue (10-100nmol/kg, *s.c.*, Novo Nordisk).

Measurement of blood pressure via carotid artery cannulation

After 5 weeks of abdominal aortic construction (AAC)-cardiac hypertrophy and heart failure, mice were anaesthetised using isoflurane (2%, Abbott laboratories, UK) in 2L/min O_2, where systemic blood pressure was monitored invasively[9]. The core temperature of the mice was maintained at 37°C with a homeothermic blanket (Havard apparatus, Cambridge, UK). Briefly, the left carotid artery was isolated and fluid-filled (heparin; 100U/ml diluted in 0.9% saline) cannula with an outer diameter of 0.7mm and internal diameter of 0.28mm (Smiths Medical-Portex, Hythe, UK) was introduced into the carotid artery. After a 5-min stabilisation period, systemic pressures were measured using PowerLab data acquisition system and LabChart 8 Pro software (ADInstruments Ltd, Oxfordshire, UK). Data was represented as an average of 10 min continuous recording.

Measurement of blood pressure via radiotelemetry

Blood pressure, heart rate and activity were measured using a radiotelemetry device (PA-C10, DSI, NL), with the catheter placed in the left carotid artery and advanced towards the aortic arch, as previously described[1, 8, 10] in C57BL/6J mice. The catheter was secured using surgical braided silk (5.0, waxed, Pearsalls sutures) and the outer wound closed with absorbable sutures (5.0, Ethicon, Johnson and Johnson) in a discontinuous pattern. The transmitter was placed *s.c.* in the right flank and the transmitter pocket was irrigated with sterile saline (0.9% saline; sodium chloride, pyrogen free). All procedures were conducted using aseptic techniques under isoflurane anaesthesia (2%, Abbott Laboratories, UK) in 2l/min O_2. Surgical anaesthesia was assessed by loss of the paw pinch reflex. Buprenorphine was administered intramuscularly (50 µg/kg, *i.m.*, Vetergesic, Alstoe Animal Health)

for pain relief. All animals were singly housed and allowed to recover for 7-10 days before recording baseline blood pressure for three 3 nights and 2 days in a quiet room. Blood pressure, heart rate activity was monitored at 10 min intervals, for duration of 2 min using the DSI software (DSI Dataquest A.R.T.) and data analysed in Microsoft Excel and GraphPad Prism 5. Data are represented as an average of 6h measurement.

Angiotensin-II murine hypertension model

Following baseline blood pressure measurement using radiotelemetry, C57BL/6J mice were implanted *s.c.* in the mid-scapular region under isoflurane anaesthesia, with osmotic pump (1002; Alzet) containing Angiotensin II (AngII, Sigma) at a dose of 1.1mg/kg/day or saline (control) for 14 days, as previously described.[7, 10] Blood pressure measurement using radiotelemetry was resumed. Mice were treated daily with vehicle (0.219M Mannitol, 5% HPCD, 1.6% ammonium acetate at pH 6.5) or αAnalogue[6] (50nmol/kg, *s.c.*, Novo Nordisk) at different time-points, as detailed below.

Experimental design 1: Animals were divided into four groups: vehicle (saline), vehicle (AngII), αAnalogue (saline) and αAnalogue (AngII). Following, saline or AngII osmotic pump infusion, mice received daily treatment of vehicle or αAnalogue (50nmol/kg, *s.c.,* Novo Nordisk) at Day 1 to Day 14 of AngII infusion (Figure 2). Total food intake (g), water consumption (ml) and body weight (g) was recorded throughout the study.

Experimental design 2: Animals were divided into two groups: vehicle (AngII) and αAnalogue (AngII). All mice received osmotic pumps containing AngII (1.1mg/kg/day) and at day 7 following AngII infusion when mice showed a significant increase in blood pressure from baseline, they received daily treatment of vehicle or αAnalogue (50nmol/kg, *s.c.,* Novo Nordisk) at Day 7 to Day 14 of AngII infusion (Figure 6).

At the end of the study (Day 15), mice were briefly anaesthetised with isoflurane (Abbott Laboratories) in 2l/min O_2 and blood was collected via cardiac puncture. Mice were culled by cervical dislocation and organs (heart and aorta) were harvested for post-analysis. Heart, lung, kidney and spleen from each mouse was excised, dried on filter paper and weighed. Organs' weights were normalised to body weight and tibia length.

Cardiac hypertrophy murine model

Mice were surgically subjected to pressure-overload induced-cardiac hypertrophy and heart failure as previously described.[11] Briefly, suprarenal aortic constriction was performed in anesthetised mice (2% isoflurane carried in O_2, Abbott Laboratories), where the abdominal aorta was ligated to the width of a 28G needle (0.36mm) using suture thread (8-0, Ethilon Sutures, Ethicon) in between the superior mesenteric and celiac bifurcation. Sham surgeries were performed in the same manner, excluding the

constriction. Post-surgery analgesia was provided in the form of buprenorphine (50 µg/kg, *i.m.*, Vetergesic). Following surgery, mice were treated with daily with vehicle (0.219M Mannitol, 5% HPCD, 1.6% ammonium acetate at pH 6.5) or αAnalogue[6] (50nmol/kg, *s.c.*, Novo Nordisk) for 5 weeks. Body weight, food and water intake were measured throughout the study. At the end of week 5, mice were briefly anaesthetised with isoflurane (Abbott Laboratories) in in 2l/min O_2 and blood was collected via cardiac puncture. Mice were culled by cervical dislocation and organs were harvested for post-analysis. Heart, lung, kidney and spleen from each mouse was excised and weighed. Organs' weights were normalised to body weight and tibia length.

Echocardiography

Anesthesia in mice was induced in a chamber with 5% isoflurane for 1 min, afterwards maintained with 1.5% on a heated platform. Imaging was performed 10 min after induction using a Vevo 2100 Imaging System with a 40-MHz linear probe (Visualsonics, Canada), as previously described.[11, 12] Cardiac dimensions as well as systolic function were assessed using M-mode imaging in long axis views. The relative wall thickness (RWT) in diastole was calculated as follows: RWT = (septal wall thickness + posterior wall thickness) / left ventricular diameter. Data analysis was performed with Vevo®2100 software v.1.2.1 (Visualsonics).

Light aversion assay

The light aversion assay is based on rodents' natural preference for darker areas compared to lighter areas[13, 14] and was used to study photophobia in rodents, a common symptom of migraine.[15] The apparatus consisted of two equal compartments: one-half (20 (d) x 20 (w) x 14 (h) cm) black plexiglass, with a black lid on top, the remaining half was transparent to enable behavioural assessment and recording. Mice were able to move freely between the two compartments through a small doorway (5 × 5 cm) in the center of the black plexiglass wall separating the two compartments, as described previously.[14] Lighting conditions (1000 lux) were established with the aid of 100-watt incandescent lamp, placed 45 cm above the centre of the box floor to the test arena and confirmed with a luxmeter (Mastech Light Meter, LX1010B, Mestech UK). At 1000 lux, light intensities in the dark zone were about 200 lux immediately inside the opening, 30 lux at the back wall across from the opening, and 10 lux at all four corners.

Mice were trained in the light/dark box under a range of lighting conditions (500-1000 lux) twice for 7 days. Mice that displayed equal preference between light/dark compartments (>40% light preference) were used in our study. Following acclimation in the testing room for 1h (200 lux), baseline recordings (10 min) were obtained, followed by systemic administration of vehicle (0.219M Mannitol, 5% HPCD, 1.6% ammonium acetate at pH 6.5) or αAnalogue (50 nmol/kg, *s.c.*) or the positive control glyceryl trinitrate (GTN, 320 nmol/kg, *i.v.*). Behavioural responses were resumed for 10min at 2h following

treatment.[16]

To evaluate the effects of chronic administration of the αAnalogue (5 weeks), sham mice were assessed on week 5 for light aversion at 1h following systemic administration of vehicle (0.219M Mannitol, 5% HPCD, 1.6% ammonium acetate at pH 6.5) or αAnalogue (50 nmol/kg, *s.c.*); behavioural responses were recorded for 10min.

Mouse behaviour was digitally recorded using an IPEVO camera (IPEVO, UK), placed 30cm above the light/dark box and attached to a laptop. Videos were continuously recorded at 30 frames/s and decomposed to individual frames of 1s interval. Each 10min experimental interval was taken to start immediately after the mouse entered the dark chamber. Behavioural experiments were conducted by a blinded observer and analysis was conducted procedurally with a custom ImageJ script, applying foreground extraction to determine the presence of the mouse against the white background of the light box floor across sequential frames. Absolute duration and % dwell time within the light and dark chambers was automatically calculated, as was the number of transitions, interpreted as crossings. Data was represented as the % of time spent in light during the 600s test session.

Glucose tolerance test

Glucose tolerance tests (GTT) were performed following a 6h fast (from 08:30 to 14:30), as previously described.[17] Glucose (1g/kg, Sigma) was injected *i.p.* and blood glucose levels were monitored for 2h (from 08:30 to 14:30) at the indicated time points after injection with a One Touch Vita glucose meter (Lifescan, UK). GTT was determined at baseline and at Day 14 following daily treatment with the αAnalogue (50nmol/kg, *s.c.*) or its respective vehicle (0.219M Mannitol, 5% HPCD, 1.6% ammonium acetate at pH 6.5).

Core body temperature measurement using radiotelemetry

Core body temperature and activity were measured using radio-telemetry as described previously.[18] Briefly, animals were anaesthetised by isoflurane (2%, Abbott Laboratories, UK) in 2l/min O_2, placed on a homeothermic heating pad and buprenorphine (50μg/kg, *i.m.*, Vetergesic, Alstoe, Animal Health, UK) was administered peri-operatively. The abdomen was shaved and scrubbed using surgical scrub and a small ventral midline abdominal incision (< 2 cm) was made to expose the abdominal muscle wall. A ventral incision was made on the abdominal wall and a small volume of (< 80 μl) sterile saline was applied to facilitate the insertion of the radio telemetry transmitter (TA10TA-F10; DSI, St Paul, Minnesota, USA). Following implantation, the abdominal wall and skin were sutured separately using absorbable sutures (Vicryl ® 4.0, Ethicon, Johnson & Johnson, UK). The mice were monitored until ambulatory and were individually housed with food and water *ad libitum*. Mice were weighed and examined daily, and were allowed a minimum 7 days post-surgical recovery period. Cages containing the telemetered animals were placed on the receiver plates (RPC-1; Data Sciences Incorporated (DSI,

Minnesota, USA); radio signals from the implanted transmitters were monitored via a fully automated data acquisition system (DataquestART, version 3.1; DSI, Minnesota, USA). Locomotor activity and core body temperature were monitored at 10 min intervals, for duration of 2 min.

Following baseline measurement for 1h, the αAnalogue (50nmol/kg, *s.c.*) and its respective vehicle (0.219M Mannitol, 5% HPCD, 1.6% ammonium acetate at pH 6.5) were administered *s.c.* and measurements were recorded for 24h thereafter.

Measurement of gene expression using Real-time reverse transcription polymerase chain reaction

Real-time reverse transcription polymerase chain reaction (RT-qPCR) was carried out as previously described.[3, 7, 10]

Total ribonucleic acid (RNA) was extracted from aorta, heart and kidney tissue using the Qiagen RNeasy Microarray tissue mini Kit (Qiagen), according to manufacturer's instructions. Total RNA (1µg) was then reverse transcribed into cDNA using the High Capacity RNA-to-cDNA Kit (Applied Biosystems, Life technologies Ltd) as per manufacturer's instructions. A thermal cycler (DNA Engine Tetrad 2 Peltier Thermal Cycler) was used for reverse transcription to complementary DNA (cDNA). Negative RT samples were carried out as a control to exclude possible contamination of genomic DNA. cDNA was then diluted 1:40 in nuclease-free water for RT-qPCR. Quantitative PCR (qPCR) was then conducted using a SYBR-green-based PCR mix (Sensi-Mix, SYBR-green No ROX; Bioline) and primers from the specific gene of interest pipetted into 100-well gene disks (Qiagen) by an automated robot (CAS1200; Qiagen Corbett), followed by PCR in a Corbett Rotor-gene 6000. Settings were as follows; initial denaturation: 10min at 95°C; cycling: 45 cycles- 10 s at 95°C, 15 s at 57°C, and 5s at 72°C; melt: 68-90°C. Samples were subjected to melting curve analysis to confirm amplification specificity. Data were collected as copies/µl and normalised against murine hypoxanthine phosphor-ribosyltransferase (HPRT), β-2-microglobulin (B_2M) and β-actin expression using GeNorm3.4 software. A list of primers are summarised in Supplemental Table I.

Western blotting

Western blot analysis was performed as previously described.[1, 2, 7] Briefly, aorta, mesentery, heart and kidney tissue was homogenised in SDS lysis buffer containing protease inhibitor (1tablet/50ml, Roche, Germany) and protein concentrations were determined. Samples were loaded onto a 7-12% Tris SDS-polyacrylamide gel electrophoresis and transferred to polyvinylidene difluoride (PVDF) membranes using a semi-dry technique (Bio-Rad). After blocking with 5% non-fat dry milk, membranes were incubated with a primary antibody (4°C, overnight), and incubated with the secondary antibody at room temperature for 1h. Proteins were detected by enhanced chemiluminescence (ECL, Piercenet) and developed using the Syngene gel doc dark room system. Densitometric analysis was performed using Image J analysis software (NIH, USA). Antibodies against eNOS (1:500, Ab5589), HO-1 (1:500,

Ab13248), nitrotyrosine (1:500, Ab61392)[2], GPX-1 (1:1000, Ab22604), CLR (1:500, Ab173562) and RAMP1 (1:1000, Ab156575) were purchased from Abcam. Antibodies against NOX-2 (gp91phox, 1:500, 611414) were obtained from BD Transduction, α-SMA (1:1000, A2547) from Sigma and klotho (1:500, KM2076) from Cosmo Bio Co. Ltd. p-p38 (1:1000, 9216) and total-p38 (1:1000, 9212) were purchased from Cell Signalling. GAPDH (1:4000, AM4300) was purchased from Life Technologies Ltd and used for a loading control. Note that for nitrosylated proteins detected using anti-nitrotyrosine antibody, densitometric analysis was conducted for the entire lane and normalised to GAPDH.

Quantification of noradrenaline using ELISA

Plasma and renal noradrenaline (NA) content were quantified using a commercially available NA ELISA kit (RE59261; IBL International, Hamburg, Germany), as previously described.[1] Briefly, kidneys were homogenised in phosphate buffered saline (1M), supplemented with protease inhibitors (Roche, Germany). Lysates were cleared by centrifugation at 2,600 *g* for 15 min at 4°C. NA content was extracted from both plasma and kidney tissue lysate and assessed by standard ELISA technique, according to the manufacturer instructions. The limit of sensitivity was 20 pg/ml and the linearity limit was 8.0 ng/ml. Cross-reactivity to other catecholamines or metabolites was manufacturer tested as <0.02%. Protein concentration of each kidney samples were determined by Bradford dye-binding method (Bio-rad) and noradrenaline content was normalised to mg of protein.

Quantification of IL-6 and TNF-α using ELISA

Briefly, frozen kidney tissues were homogenised in phosphate buffered saline (1M), supplemented with protease inhibitors (Roche, Germany). Lysates were cleared by centrifugation at 2,600 *g* for 15 min at 4°C. Renal interleukin-6 (IL-6) and Tumour necrosis factor-α (TNF-α) content were quantified using a commercially available ELISA kit (RAB0308 & RAB0477, Sigma, UK), following manufacturer's protocol. Protein concentration of each kidney samples were determined by Bradford dye-binding method (Bio-rad). The final concentrations of both IL-6 and TNF-α were calculated with respect to the total amount of protein (mg).

Histology

Aorta, heart and kidney tissues were fixed in 4% paraformaldehyde (overnight, 4°C), as previously described.[7, 19, 20] For heart and kidney samples, after routine paraffin wax embedding, transverse sections (6μm) were prepared using a Reichert-Jung 2030 Biocut microtome onto poly-L-lysine slides. For aorta samples, following fixation aorta segments were embedded in optimal cutting temperature compound (OCT, VWR), frozen on dry ice in a cryomould and cryosectioned on a microtome cryostat (6μm, Bright, UK) on Superfrost Plus glass slides. Sections were allowed to dry overnight. Approximately 8-10 transverse sections were obtained from each heart or aorta per mouse for staining.

Aorta Staining: Sections of aorta were stained with Masson's trichrome, as previously described[7, 21]. All slides were imaged using an Olympus Colourview III camera, connected to an Olympus BX51 microscope (x10 magnification). Images were taken using CellSens Dimension viewing software (Version 1.1.3, Olympus). Aortic wall width (medial thickness) were measured (mean of 8 measurements taken from at least 3 sections for each 4 mice/group) using ImageJ analysis software (scale bars, 100μm; NIH, USA).

Heart Staining: Picrosirius Red (0.1% w/v) staining was used to visualise collagen fibres in the heart[20] and fibrosis was assessed blindly. All slides were imaged using a Leica Diaplan microsope (x10 magnification) and images captured using ProgRes Capture Pro viewing software (Version 2.8.8, JENOPTIK) under bright light and circular polarised light according to a modified Junqueira method.[22, 23] Cardiac fibrosis was quantified on circular polarised light images using Image J analysis software (scale bars, 200μm; NIH, USA).

Conjugated wheat germ agglutinin (WGA, CF488A, Biotium) staining was applied to outline cardiomyocyte boundaries and quantify cross-sectional area.[20] All slides were imaged using an Olympus Colourview III camera, connected to an Olympus BX51 microscope (x40 magnification). Images were taken using CellSens Dimension viewing software (Version 1.1.3, Olympus). Myocyte cross-sectional area was employed as an index of cardiac hypertrophy and determined on fibres with circular shapes in a blinded fashion by quantitative image analysis using Image J (scale bars, 20μm; NIH, USA).

Capillaries were immunostained with isolectin B4 (Vector B-1205) and capillary density was quantified as the number of capillaries per mm^2 of LV sections[11]. All slides were imaged using an Olympus Colourview III camera, connected to an Olympus BX51 microscope (x40 magnification). Images were taken using CellSens Dimension viewing software (Version 1.1.3, Olympus). Number of capillaries were manually counted in a blinded fashion by quantitative image analysis using Image J (scale bars, 20μm; NIH, USA).

Apoptosis was detected by using terminal deoxyribonucleotide transferase (TdT)-mediated dUTP nick-end labelling (TUNEL) staining (Millipore S7111 kit) in LV sections[24]. All slides were imaged using an Olympus Colourview III camera, connected to an Olympus BX51 microscope (x20 magnification). Images were taken using CellSens Dimension viewing software (Version 1.1.3, Olympus). TUNEL-positive myocytes and DAPI-stained nuclei were manually counted by quantitative image analysis using Image J (scale bars, 50μm; NIH, USA).

Kidney Staining: Sections of kidney were stained with Periodic acid–Schiff's (PAS) and counter stained with hematoxylin, as previously described[25]. All slides were imaged using an Olympus Colourview III camera, connected to an Olympus BX51 microscope (x40 magnification). Images were taken using

CellSens Dimension viewing software (Version 1.1.3, Olympus). A semi quantitative system was used to grade mesangial expansion by either diffuse (irregular streaks) or segmental (localized to a lobule) type. In each of the experimental animals, 20 glomeruli were examined for mesangial expansion and each glomerulus was graded 0 to 4 corresponding to the percentage of mesangial matrix covering/occupying the urinary space of the Bowman's capsule. Glomeruli were assessed and graded 1-4, as follows: grade 1, involvement of 0-24%; grade 2, 25-29%; grade 3, 50-74% or grade 4; 75-100% of mesangial area to Bowman's capsule (scale bars, 20μm; NIH, USA). Mean glomerular score index was determined for each kidney[26].

All morphometric measurements were performed in a blinded manner.

Statistical analysis

Data in the manuscript is expressed as mean ± SEM. Statistical analysis was performed using a two-tailed Student's *t*-test (2 unpaired groups), one-way or repeated measures two-way analysis of variance (ANOVA) followed by Bonferroni's comparison *post hoc* test (multiple groups comparison). $p<0.05$ was considered to represent a significant difference.

Supplemental Tables

Supplemental Table 1. Gene primer sequences

Target Gene	Primer Sequence	Accession number
**Akt**	F: CGTCGCCAAGGATGAGGTTG<br>R: GTCGTGGGTCTGGAATGAGT	NM_001165894.1
**α-MHC**	F: GCTGGAAGAAAAGCTCAAGAAGAAA<br>R: TCTCTATCTGCACGGATGTGG	NM_001164171.1
**β-MHC**	F: CACCTACCAGACAGAGGAAGA<br>R: GGAGCTGGGTAGCACAAGA	NM_080728.2
**α-skeletal actin**	F: CTAAATCCAAGTCCTGCAAGTG<br>R: ACATGGTGTCTAGTTTCAGAGG	NM_001272041.1
**ANP**	F: GGATTTCAAGAACCTGCTAGACC<br>R: GCAGAGCCCTCAGTTTGCT	NM_008725.3
**B_2M**	F: GTCGCTTCAGTCGTCAGCA<br>R: TTGAGGGGTTTTCTGGATAGCA	NM_009735.3
**Bcl-2**	F: AGGCTGGGATGCCTTTGTGG<br>R: TGTTTGGGGCAGGTTTGTCG	NM_009741.5
**BNP**	F: TGGGCTGTAACGCACTGAA<br>R: TGTTGTGGCAAGTTTGTGCTT	NM_001287348.1
**Collagen Type 1 α1**	F: TCTGACTGGAAGAGCGGAGAG<br>R: AGACGGCTGAGTAGGGAACA	NM_007742.4
**Collagen Type 1 α2**	F: TGGATACGCGGACTCTGTTG<br>R: CCCTTTCGTACTGATCCCGATT	NM_007743.2
**Collagen Type 3 α1**	F: GGGAGGAATGGGTGGCTATC<br>R: CTGGGCCTTTGATACCTGGA	NM_009930.2
**Collagen Type 4 α1**	F: CTGGAGAAAAGGGCCAGAT<br>R: TCCTTAACTTGTGCCTGTCC	NM_009931.2
**CTGF**	F: GGGCCTCTTCTGCGATTC<br>R: ATCCAGGCAAGTGCATTGGTA	NM_010217.2
**Cystatin C**	F: GAGTACAACAAGGGCAGCAAC<br>R: AGCAGAGTGCCTTCCTCATCA	NM_009976.4
**eNOS**	F: GACCCTCACCGCTACAA AT<br>R: GTCCTGGTGTCCAGATCCAT	NM_008713.4
**Fibronectin**	F: CCGGTGGCTGTCAGTCAGA<br>R: CCGTTCCCACTGCTGATTTATC	NM_006495700.2
**GPX-1**	F: TTCGGACACCAGGAGAATGG<br>R: TAAAGAGCGGGTGAGCCTTC	NM_008160.6
**HIF-1α**	F: CGAGAACGAGAAGAAAAAGATGAGT<br>R: CGTAAATAACTGATGGTGAGCCT	NM_010431.2
**HO-1**	F: CAACATTGAGCTGTTTGAGGAG<br>R: CTCTGACCAAGTGACGCCAT	NM_010442.2
	F: CCTGGTTCATCATCGCTAATC	NM_013556.2

**HPRT**	R: TCCTCCTCAGACCGCTTTT	
**MMP-2**	F: GACAAGTTCTGGAGATACAATGAAGTG<br>R: CAGGTTATCAGGGATGGCATTC	NM_006530751.1
**NGAL**	F: AATGTCACCTCCATCCTGGTCA<br>R: GACAGCTCCTTGGTTCTTCCATAC	NM_008491.1
**NF-κB**	F: CCTACGGAACTGGGCAAATGT<br>R: TCCCCTCTGTTTTGGTTGCT	NM_ 008689.2
**NOX-2**	F: ACTCCTTGGGTCAGCACTGG<br>R: GTTCCTGTCCAGTTGTCTTCG	NM_007807.5
**NQO1**	F: TCATTCTCTGGCCGATTCA<br>R: TGCTGTAAACCAGTTGAGGTTC	NM_008706.5
**Osteopontin**	F: AAACCAGCCAAGGTAAGCCT<br>R: GCAAAAGCAATCACTGCCA	NM_001204201.1
**p53**	F: ATGCCCATGCTACAGAGGAG<br>R: AGACTGGCCCTTCTTGGTCT	NM_001127233.1
**RANTES**	F: TGCTCCAATCTTGCAGTCGT<br>R: GCGTATACAGGGTCAGAATCAAG	NM_013653.3
**SERCA-2**	F: TGGAACCTTTGCCGCTCATTT<br>R: CAGAGGCTGGTAGATGTGTT	NM_009722.3
**TGF-β_1**	F: TCAGACATTCGGGAAGCAGT<br>R: GCCCTGTATTCCGTCTCCTTG	NM_011577.2
**TIMP-2**	F: GATTCAGTATGAGATCAAGCAGATAAAGA<br>R: GCGAGACCCCGCACACT	NM_011594.3
**α-SMA**	F: ACTACTGCCGAGCGTGA<br>R: ATAGGTGGTTTCGTGGATGC	NM_007392.3
**β-actin**	F: CACAGCTTCTTTGCAGCTCCTT<br>R: TCAGGATACCTCTCTTGCTCT	NM_007393.5

Supplemental Table 1. List of gene primer sequences used for qRT-PCR studies.

Supplemental Table 2: Changes in organ weight in AngII and saline-infused mice pre-treated with αAnalogue or vehicle

Organs	Vehicle		αAnalogue	
	Saline	AngII	Saline	AngII
Total Heart:Body Weight (mg/g)	4.4 ± 0.3	6.3 ± 0.2 ***	4.5 ± 0.2	5.2 ± 0.2 ††
Total Heart:Tibia length (mg/mm)	7.9 ± 0.4	10.0 ± 0.4 ***	7.5 ± 0.2	8.4 ± 0.3 ††
Left Ventricle:Body Weight (mg/g)	3.4 ± 0.2	4.9 ± 0.1 ***	3.5 ±0.2	4.2 ± 0.1 †††
Lung Oedema (Wet:Dry Ratio)	4.2 ± 0.1	4.2 + 0.1	4.1 ± 0.1	4.1 + 0.1
Kidney:Body Weight (mg/g)	5.8 ± 0.3	5.8 ± 0.3	5.8 ± 0.3	5.7 ± 0.2
Kidney:Tibia length (mg/mm)	10.8 ± 0.3	9.1 ± 0.4	9.4 ± 0.2	9.4 ± 0.6
Spleen:Body Weight (mg/g)	2.5 ± 0.2	3.1 ± 0.2	2.7 ± 0.2	3.1 ± 0.3
Spleen:Tibia length (mg/mm)	4.7 ± 0.2	4.8 ± 0.3	4.5 ± 0.3	5.1 ± 0.4

Supplemental Table 2. Changes in organ weight in the heart of AngII and saline-infused mice treated daily with α-CGRP analogue (αAnalogue, 50nmol/kg) or vehicle (*s.c.*) daily for 14 days. Mice were implanted with osmotic pumps containing saline or AngII (1.1mg/kg/day) and treated daily with vehicle or αAnalogue (50nmol/kg, *s.c.*) for 14 days (n=6-9). At day 15, mice were sacrificed, organs were weighed and normalised to body weight (mg/g) or tibia length (mg/mm). Results show mean ± SEM. ***p<0.001 vs vehicle-treated saline-infused mice; ††p<0.01, †††p<0.001 vs vehicle-treated AngII-infused mice (2-way ANOVA + Bonferroni *post hoc* test).

Supplemental Table 3: Changes in gene expression in the heart of AngII and saline-infused mice pre-treated with αAnalogue or vehicle				
	Vehicle		αAnalogue	
**Gene**	**Saline**	**AngII**	**Saline**	**AngII**
CTGF	89.3 ± 14.6	220.3 ± 44.0 *	152.0 ± 42.9	67.6 ± 20.1 †
Akt	499.1 ± 80.1	721.8 ± 96.6	666.7 ± 78.6	365.7 ± 59.3 †
TIMP-2	337.6 ± 22.5	396.2 ± 33.5	391.5 ± 52.1	223.5 ± 30.0 *, ††
NF-κB	231.6 ± 16.5	263.6 ± 25.2	276.9 ± 14.6	185.3 ± 12.7 **, †
Bcl-2	16.0 ± 1.5	17.3 ± 2.0	13.5 ± 2.1	12.7 ± 1.7
p53	5082 ± 569.4	7267 ± 543.1 *	6475 ± 518.5	4926 ± 359.5 ††

Supplemental Table 3. Changes in gene expression in in the heart of AngII and saline-infused mice treated daily with α-CGRP analogue (αAnalogue, 50nmol/kg) or vehicle (*s.c.*) daily for 14 days. Mice implanted with osmotic pumps containing saline (S) or AngII (A, 1.1mg/kg/day) and treated daily with vehicle or αAnalogue (50nmol/kg, s.c.) for 14 days. mRNA expression measured by qRT-PCR (n=5-11) for connective tissue growth factor (CTG), Protein Kinase B (PKB, Akt), tissue inhibitor of metalloproteinase-2 (TIMP-2), nuclear factor kappa B cells (NF-κB), apoptosis regulator B-cell lymphoma 2 (Bcl-2) and apoptotic marker p53 in the mice heart. Results expressed as copy numbers per µl normalised to HPRT, B_2M and β-actin. Results show mean ± SEM. *p<0.05 vs vehicle-treated saline-infused mice; †p<0.05, ††p<0.01 vs vehicle-treated AngII-infused mice (2-way ANOVA + Bonferroni *post hoc* test).

Supplemental Table 4. Changes in gene expression in the aorta of vehicle and αAnalogue treated AngII-infused mice		
	**AngII**	
**Gene**	**Vehicle**	**αAnalogue**
Vascular dysfunction		
eNOS	210.2 ± 14.4	162.2 ± 31.5
Akt	663.3 ± 23.6	567.9 ± 30.1 *
Remodelling and fibrosis		
TGF-β	3282.2 ± 395.3	1567.4 ± 412.6 *
CTGF	2957.2 ± 270.3	1513.3 ± 412.6 *
COL1*A1*	8869.7 ± 2537.2	5253.2 ± 1553.8
COL3*A1*	2765.6 ± 802.5	1139.5 ± 325.45
α-SMA	5288.1 ± 343.5	3272.2 ± 271.6 **
Vascular Inflammation		
RANTES	2524.6 ± 1132.0	928.9 ± 228.8
Osteopontin	36.0 ± 10.9	4.8 ± 2.4 *
Oxidative Stress		
HO-1	1169.0 ± 349.0	226.3 ±68.2 *
NOX-2	52.8 ± 1.4	32.3 ± 2.8 **

Supplemental Table 4. Changes in gene expression in the aorta of vehicle and α-CGRP analogue (αAnalogue) treated Angiotensin II (AngII)-infused mice. Mice infused with AngII (1.1mg/kg/day) and treated daily with vehicle or αAnalogue (50nmol/kg, *s.c.*) at day 7 to 14. mRNA expression measured by qRT-PCR (n=4) for endothelial nitric oxide (eNOS), Protein kinase B (PKB, Akt), transforming growth factor beta-1 (TGF-β1), connective tissue growth factor (CTGF), collagen type 1 α1 (COL*1A1*), collagen type 3 α1 (COL*3A1*), alpha-smooth muscle actin (α-SMA), RANTES, osteopontin, haem-oxygenase 1 (HO-1), NAD(P)H dehydrogenase, quinone-1 (NQO1) and NADPH oxidase-2 (NOX-2) in the mice aorta. Results expressed as copy numbers per μl normalised to HPRT, B2M and β-actin. Results are shown as mean ± SEM. *p<0.05, **p<0.01 vs vehicle-treated AngII-infused mice (two-tailed Student *t*-test).

Supplemental Table 5: Changes in organ weight in αAnalogue or vehicle-treated AngII-induced hypertensive mice

Organs	AngII	
	Vehicle	αAnalogue
Total Heart:Body Weight (mg/g)	6.3 ± 0.5	4.9 ± 0.2 *
Total Heart:Tibia length (mg/mm)	9.4 ± 0.4	7.5 ± 0.6 *
Left Ventricle:Body Weight (mg/g)	5.2 ± 0.4	3.9 ± 0.1 *
Lung Oedema (Wet:Dry Ratio)	3.7 ± 0.2	3.9 ± 0.3
Kidney:Body Weight (mg/g)	5.8 ± 0.3	5.3 ± 0.3
Kidney:Tibia length (mg/mm)	8.4 ± 0.2	8.2 ± 0.1
Spleen:Body Weight (mg/g)	2.6 ± 0.3	2.9 ± 0.2
Spleen:Tibia length (mg/mm)	3.8 ± 0.3	4.5 ± 0.6

Supplemental Table 5. Changes in organ weight in the heart of α-CGRP analogue (αAnalogue, 50nmol/kg) or vehicle (*s.c.*)-treated AngII-infused mice. Mice were infused with AngII (1.1mg/kg/day) osmotic pumps for 14 days and treated with vehicle or αAnalogue (50nmol/kg) on Day 7-14 (n=4). At day 15, mice were sacrificed, organs were weighed and normalised to body weight (mg/g) or tibia length (mg/mm). Results show mean ± SEM. *p<0.05 vs vehicle-treated AngII-infused mice (two-tailed Student t-test).

Supplemental Table 6: Changes in gene expression in the heart of vehicle and αAnalogue treated AngII-infused mice		
	**AngII**	
**Gene**	**Vehicle**	**αAnalogue**
Remodelling and fibrosis		
CTGF	1118 ± 85.6	719.0 ± 206.3
Fibronectin	586.0 ± 62.0	330.6 ± 87.6
COL1*A1*	825.5 ± 140.0	569.3 ± 157.8
COL3*A1*	852.2 ± 140.0	282.5 ± 91.9 *
COL4*A1*	4670.9 ± 415.7	4108.7 ± 512.0
SERCA-2	189.2 ± 13.5	163.7 ± 18.7
ANP	40224 ± 7485.1	6026.7 ± 2559.2 **
BNP	1365.6 ± 220.9	1517.8 ± 641.2
Oxidative Stress		
NOX-2	67.7 ± 7.1	44.4 ± 3.9 *

Supplemental Table 6. Changes in gene expression in the heart of vehicle and α-CGRP analogue (αAnalogue) treated Angiotensin II (AngII)-infused mice. Mice infused with AngII (1.1mg/kg/day) and treated daily with vehicle or αAnalogue (50nmol/kg, s.c.) at day 7 to 14 (n=4). mRNA expression measured by qRT-PCR for connective tissue growth factor (CTGF), fibronectin, collagen type 1 α1 (COL*1A1*), collagen type 3 α1 (COL*3A1*), collagen type 4 α1 (COL*4A1*), sarcoplasmic reticulum Ca2+ ATPase-2 (SERCA-2), atrial natriuretic peptide (ANP), brain natriuretic peptide (BNP) and NADPH oxidase-2 (NOX-2) in heart. Results expressed as copy numbers per μl normalised to HPRT, B2M and β-actin. Data represent mean + S.E.M. *p<0.05, **p<0.01 vs vehicle-treated AngII-infused (two-tailed Student t-test).

Supplemental Table 7. Echocardiographic parameters of left ventricular size and function 5 weeks post AAC-induced hypertrophy and heart failure in mice treated with αAnalogue or vehicle

Parameters	Vehicle		αAnalogue	
	Sham	AAC	Sham	AAC
n	8	6	8	8
HR [bpm]	430.3 ± 11.59	455.5 ± 36.49	452.9 ± 10.37	457.9 ± 17.49
LVID;d [mm]	4.18 ± 0.10	4.36 ± 0.13	4.10 ± 0.11	4.32 ± 0.07
LVID;s [mm]	3.16 ± 0.08	3.68 ± 0.21*	3.01 ± 0.09	3.32 ± 0.09
LVV;d [µl]	40.14 + 2.43	59.04 + 7.82**	35.76 + 2.80	45.13 + 3.04
LVV;s [µl]	78.41 ± 4.39	86.44 ± 5.97	75.00 ± 4.98	84.61 ± 3.16
septW [mm]	0.78 + 0.03	1.02 + 0.04 ***	0.79 + 0.03	0.87 + 0.03 ††
postW [mm]	0.74 + 0.03	1.03 + 0.06 ***	0.71 + 0.03	0.78 + 0.02 †††
rWT	0.37 + 0.02	0.47 + 0.02 **	0.37 + 0.02	0.38 + 0.01 ††
SV [µl]	38.27 + 2.30	27.40 + 2.7 **	39.24 + 2.43	39.48 + 1.34 ††
EF [%]	48.80 + 1.17	33.10 + 5.17 ***	52.51 + 1.24	46.87 + 1.96 ††
FS [%]	24.39 + 0.72	15.91 + 2.92 ***	26.67 + 0.79	23.37 + 1.17 ††

Supplemental Table 7. Echocardiographic parameters of left ventricular size and function 5 weeks post AAC-induced hypertrophy and heart failure. Mice were treated daily with vehicle or α-CGRP analogue (αAnalogue, 50nmol/kg/day, *s.c.*) post surgery (n=6-8). (HR: heart rate, LVID;d: left ventricular dimension in diastole, LVID;s: left ventricular dimension in systole, LVV;d: left ventricular volume in diastole, LVV;s: left ventricular volume in systole, septW: septal wall thickness, postW: posterior wall thickness, rWT: relative wall thickness, SV: stroke volume, EF: ejection fraction, FS: fractional shortening. Data represented as mean ± SEM (n=6-8). *$p<0.05$, **$p<0.01$, ***$p<0.001$ vs respective sham-treated; ††$p<0.01$, †††$p<0.001$ vs vehicle-treated AAC (2-way ANOVA + Bonferroni *post hoc* test).

Supplemental Table 8: Changes in organ weight in AAC and sham-treated WT mice pre-treated with αAnalogue or vehicle

Organs	Vehicle		αAnalogue	
	Sham	AAC	Sham	AAC
Total Heart:Body Weight (mg/g)	4.7 ± 0.1	9.1 ± 0.1***	4.7 ± 0.2	5.9 ± 0.3†††
Total Heart:Tibia length (mg/mm)	7.6 ± 0.2	13.8 ± 1.2***	7.8 ± 0.3	9.6 ± 0.5†††
LV:Body Weight (mg/g)	3.5 ± 0.1	7.1 ± 0.6***	3.5 ± 0.1	4.5 ± 0.3*,†††
Lung Oedema (Wet:Dry Ratio)	4.3 ± 0.0	4.5 ± 0.3	4.2 ± 0.1	4.3 ± 0.1
Dry Lung:Tibia length (mg/g)	1.7 ± 0.1	3.8 ± 0.8***	2.0 ± 0.1	1.9 ± 0.1††

Supplemental Table 8. Changes in organ weight in α-CGRP analogue (αAnalogue, 50nmol/kg) or vehicle (*s.c.*)-treated sham or AAC-induced cardiac hypertrophy and heart failure mice. Mice were treated daily for 5 weeks post surgery. Organs were weighed and normalised to body weight (mg/g) or tibia length (mg/mm). Results show mean + SEM (n=6-8). *p<0.05, ***p<0.001 vs respective sham-treated; ††p<0.01, †††p<0.001 vs vehicle-treated AAC mice (2-WAY ANOVA + Bonferroni *post hoc* test).

Supplemental Figures

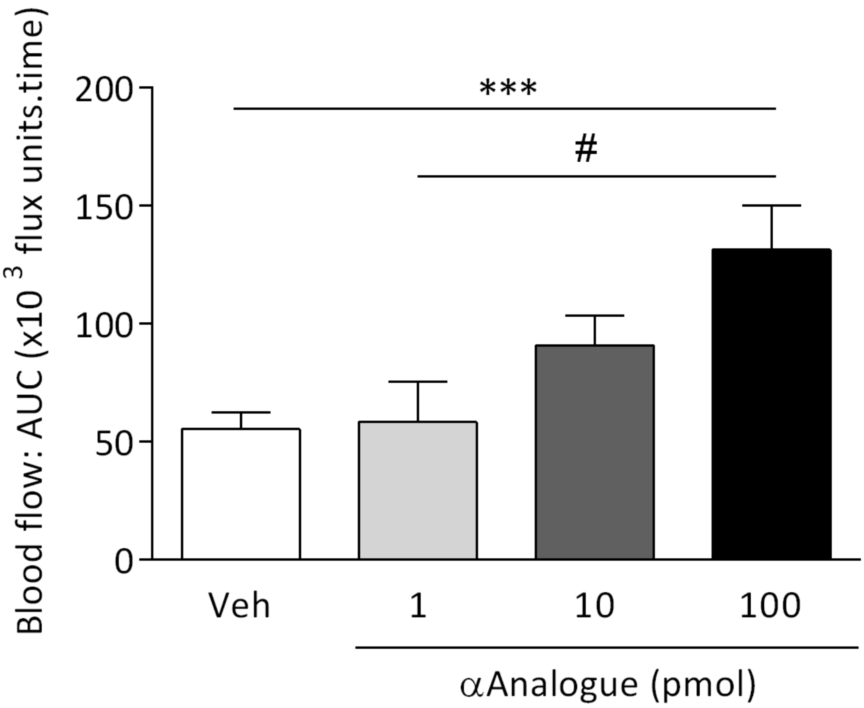

Supplemental Figure 1. Effect of local administration α-CGRP analogue (αAnalogue) on vascular skin blood flow in naïve mice. Blood flow was monitored using Full-field Laser Perfusion Imager (FLPI) in the ear skin of anaesthetised mice (n=4-6) at baseline (5 min) and following intradermal injection of αAnalogue (1-100pmol) in the ipsilateral ear and vehicle (0.219M Mannitol, 5% HPCD, 1.6% ammonium acetate at pH 6.5) in the contralateral ear. Results show mean $\pm$ SEM for area under the curve (AUC) for the 30 min recording. ***p<0.001 vs vehicle-treated ear; #p<0.05 vs αAnalogue treated ear (One-way ANOVA + Bonferroni *post hoc* test).

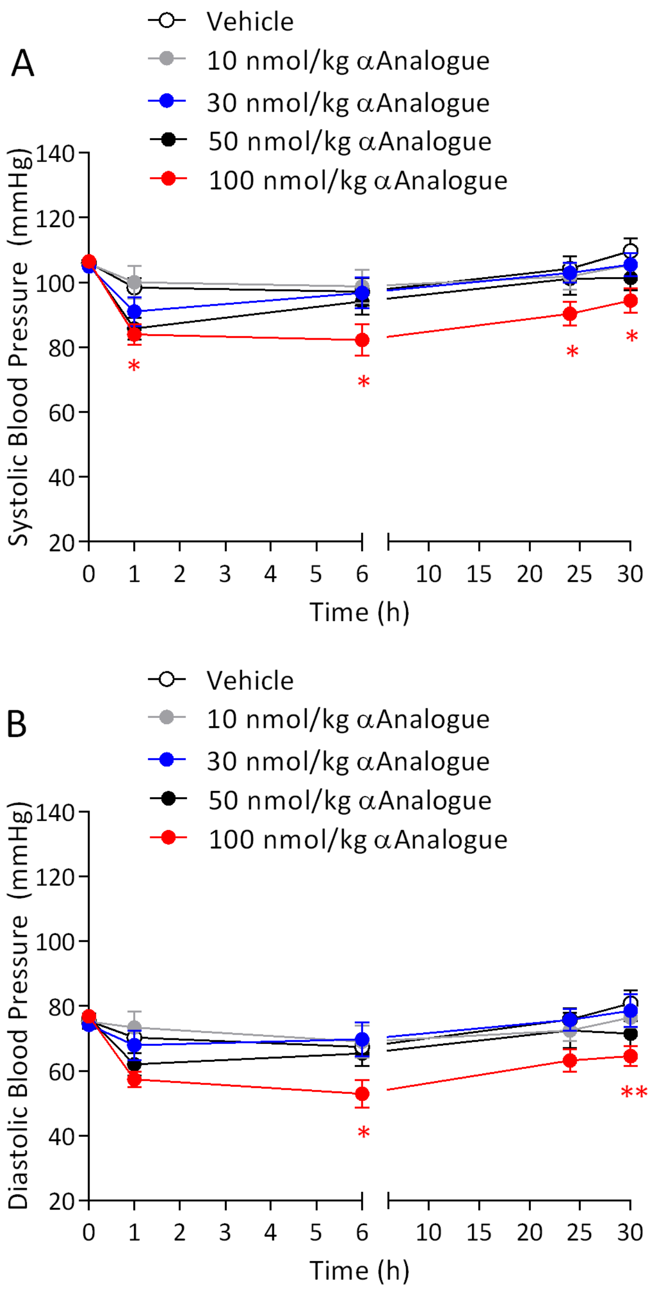

Supplemental Figure 2. Effect of systemic administration of α-CGRP analogue (αAnalogue) on blood pressure in naïve mice. (A) Systolic and **(B)** diastolic blood pressure was monitored using tail-cuff plethysmography in trained conscious restrained mice at baseline and 1, 6, 24 and 30h following vehicle (0.219M Mannitol, 5% HPCD, 1.6% ammonium acetate at pH 6.5) or αAnalogue administration (10, 30, 50 or 100nmol/kg, *s.c.*). Blood pressure values were obtained for each animal and results show mean ± SEM for each group of mice (n=7). *$p<0.05$, **$p<0.01$ vehicle vs 100nmol/kg αAnalogue treatment (Repeated measures 2-way ANOVA + Bonferroni *post hoc* test).

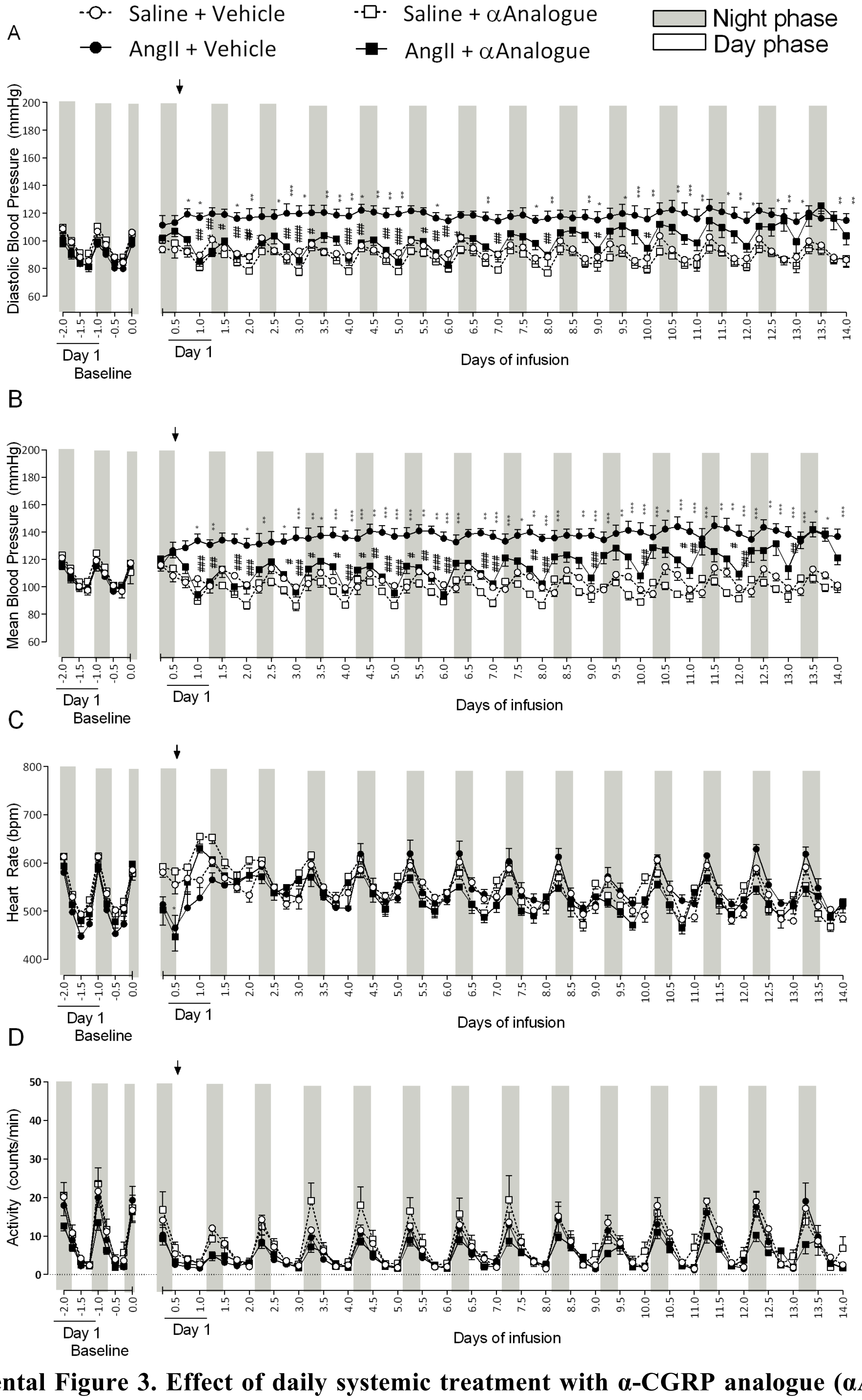

Supplemental Figure 3. Effect of daily systemic treatment with α-CGRP analogue (αAnalogue, 50nmol/kg) on cardiovascular haemodynamics in angiotensin II (AngII)-induced hypertension. Mice were infused with either AngII (1.1mg/kg/day) or control (saline) with osmotic pumps for 14 days and treated daily with vehicle or αAnalogue (50nmol/kg, *s.c.*). (**A**) Diastolic blood pressure, (**B**) mean blood pressure, (**C**) heart rate and (**D**) activity were measured by radiotelemetry. Results show measurement taken every 10 min, expressed as 6h average. Mice experience a 12/12h light/dark cycle, with the dark cycle shown in the grey striped area. Arrow represents the start of daily treatment. Results show mean ± SEM (n=4-7). *p<0.05, **p<0.01, ***p<0.001 vs vehicle-treated saline-infused mice; #p<0.05, ##p<0.01, ###p<0.001 for αAnalogue-treated mice AngII-infused vs vehicle-treated AngII-infused mice (Repeated measures 2-way ANOVA + Bonferroni *post hoc* test).

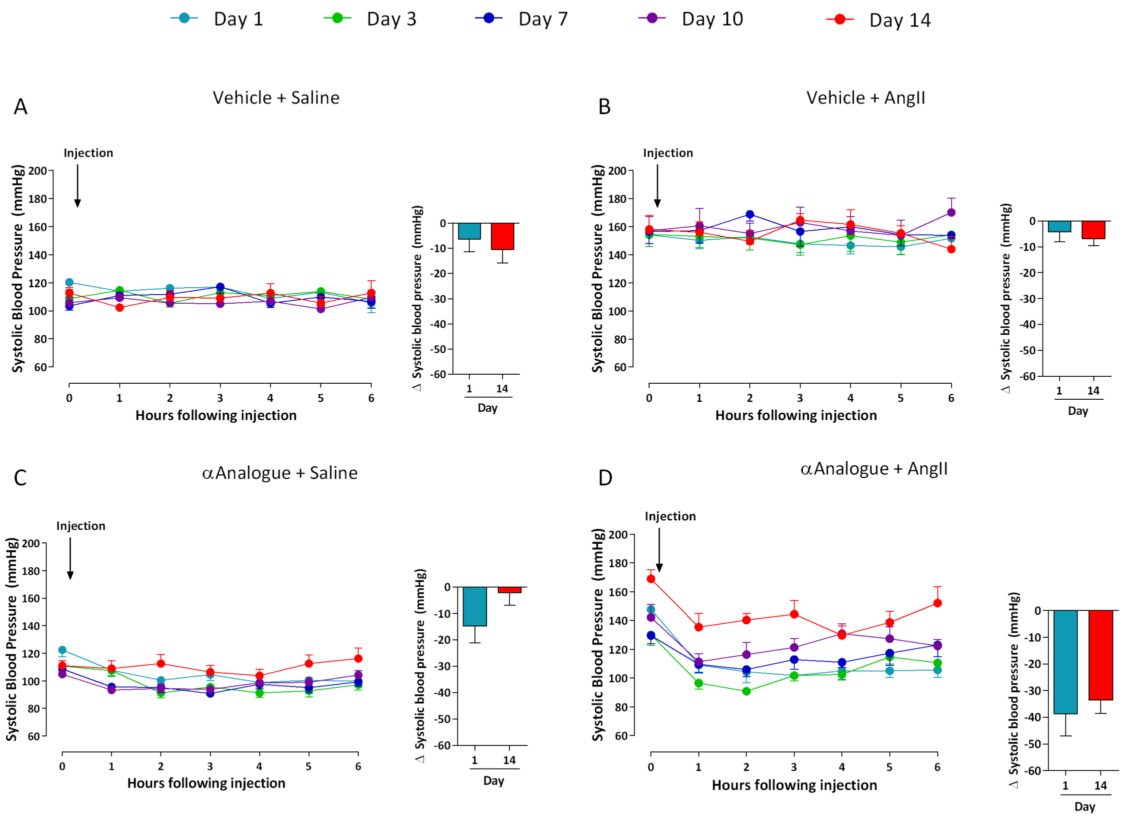

Supplemental Figure 4. Daily systemic treatment with the α-CGRP analogue (αAnalogue) produces a reproducible reduction in blood pressure in Angiotensin II (AngII)-induced hypertension in mice. 6h time course profile of systolic blood pressure following injection of vehicle or αAnalogue (50nmol/kg) at Day 1, 3, 7, 10 and 14 in mice infused with saline **(A and C)** or AngII **(B and D)**. **Left Panels,** Detailed analysis of SBP changes pre-and up to 6h post- αAnalogue or vehicle injection in mice infused with saline or AngII. **Right Panels,** Change (Δ) in SBP at Day 1 and Day 14 at 1h following injection from baseline (n=4-7). Results show mean ± SEM.

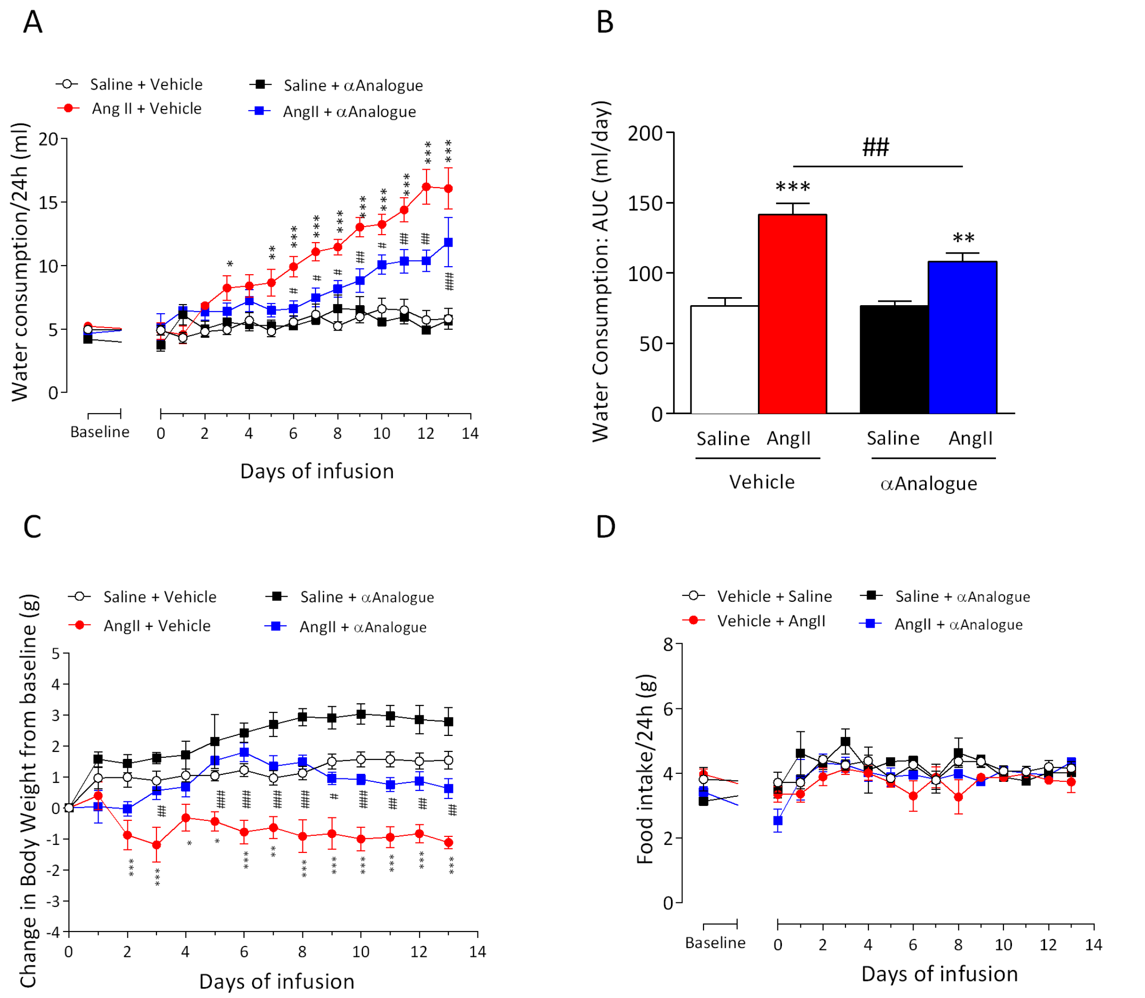

Supplemental Figure 5. Effects of the α-CGRP analogue (αAnalogue) or vehicle on (A-B) water consumption, (C) body weight and (D) food intake in Angiotensin II (AngII)-induced hypertension in mice. Baseline measurements of body weight, food and water intake were taken for 7 days prior to osmotic pump implantation. Mice were administered with osmotic pumps containing saline or AngII (1.1 mg/kg/day for 14 day). Measurements were taken daily. Data are mean ± S.E.M from n=5-7. AUC represents area under the curve results throughout the 14 day recording. Results show mean ± SEM. *p<0.05, **p<0.01, ***p<0.001 versus vehicle-treated group, #p<0.05, ##p<0.01, ###p<0.001 versus αAnalogue-treated group (Repeated measures 2-way ANOVA + Bonferroni *post hoc* test for A,C-D and 2-way ANOVA + Bonferroni *post hoc* test for B).

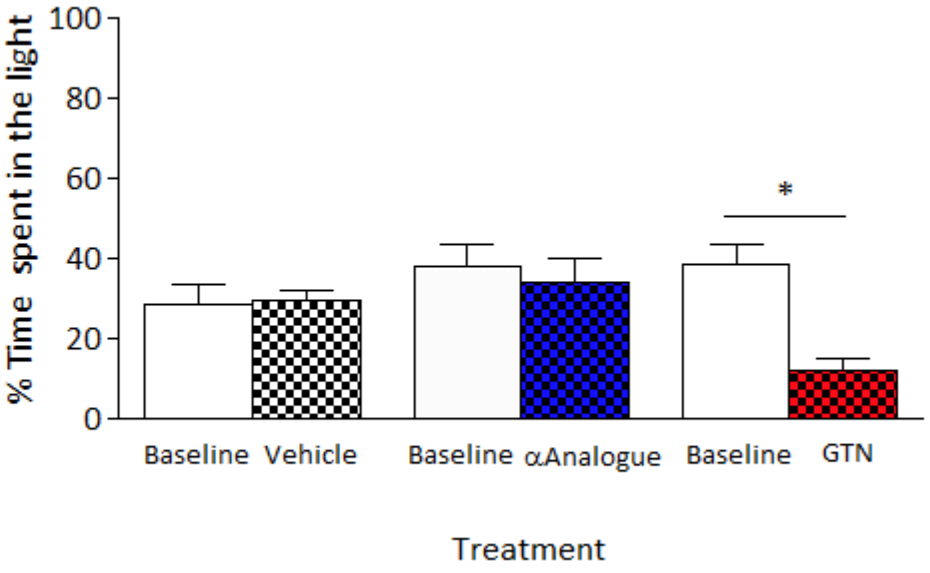

Supplemental Figure 6. Effects of the α-CGRP analogue (αAnalogue) on light-aversion. Mice were trained twice daily for 5 days to enable an even distribution to enter the dark covered zone or bright light uncovered area (n=6-7). On the test day, time (s) spent in the light (1000 lux) was recorded for 600s at baseline and 1h following administration of αAnalogue (50nmol/kg, *s.c.*) or glyceryl trinitrate (GTN, 352nmol/kg, *i.v.*). Results show % time spent in light in mice, mean $\pm$ SEM (n=6-7). *$p<0.05$ vs respective baseline (paired t-tests).

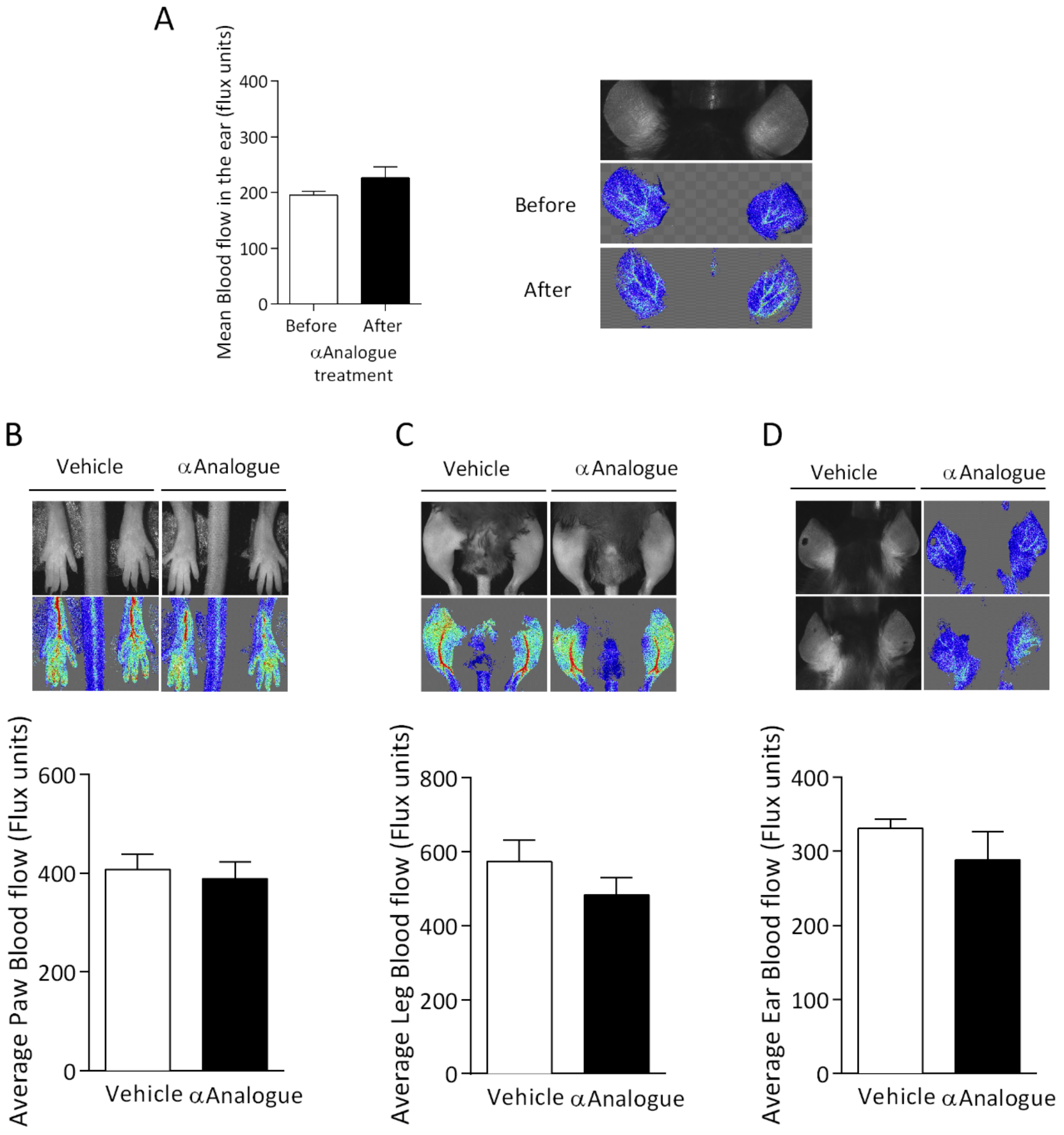

Supplemental Figure 7. Effects of the α-CGRP analogue (αAnalogue) on peripheral blood flow in mice. Mice were briefly anaesthetised with isoflurane (2%) and blood flow was monitored using Full-field Laser Perfusion Imager (FLPI) for 5 min. **(A)** Average blood flow in the ear vasculature at baseline (before) and 1h following a single treatment of αAnalogue (50nmol/kg, *s.c.*) in mice (n=6). Average blood flow in the **(B)** paw, **(C)** leg and **(D)** ear of mice at day 14 following daily treatment of vehicle or αAnalogue (50nmol/kg, *s.c.*). Representative FLPI pictures alongside grey/black 'photo' showing blood flow for vehicle and αAnalogue treatment. Results represent mean ± SEM for average measurement (flux units) for 5 min recording. p>0.05 ns (two-tailed Student's *t*-test).

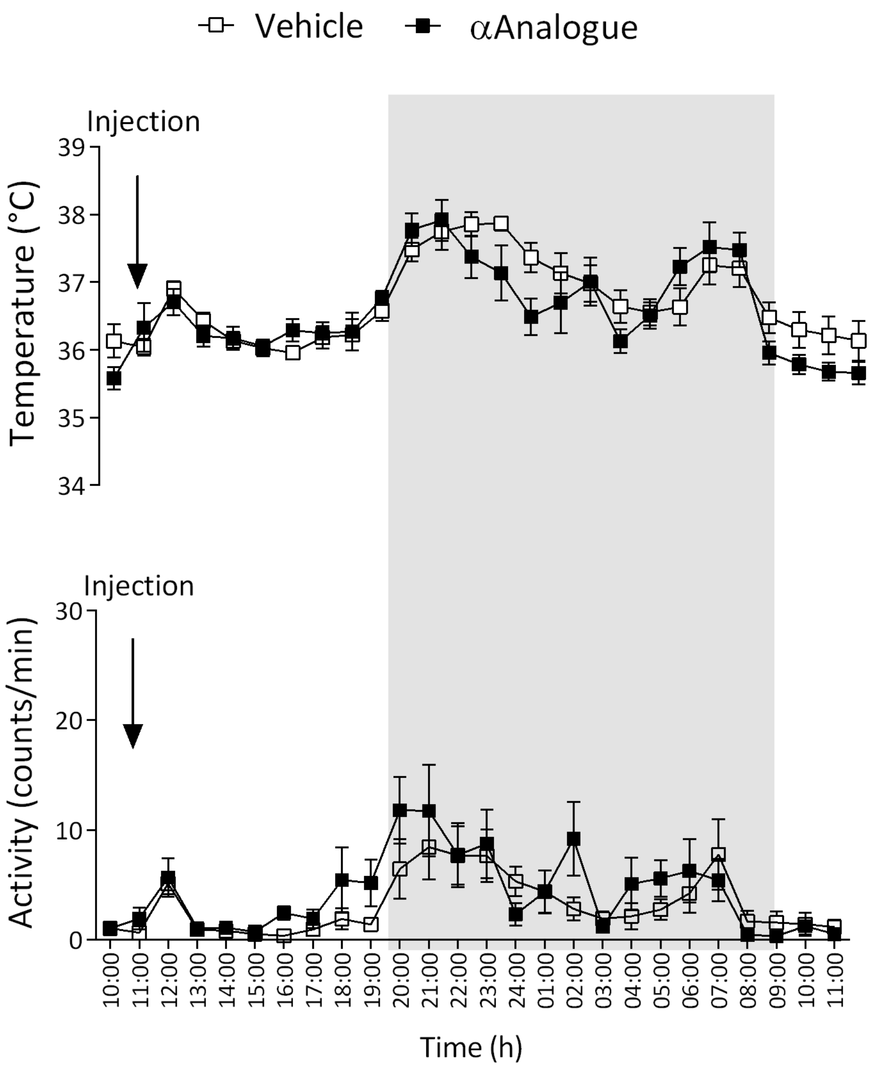

Supplemental Figure 8. Effects of α-CGRP analogue (αAnalogue) on core body temperature and activity in mice Core body temperature and activity recordings over 24h at baseline and following vehicle or αAnalogue (50nmol/kg, *s.c.*) treatment in conscious mice (n=5). Results represent average 1h recording, mean $\pm$ SEM. Arrow denotes time of treatment and grey area represents night/dark period. NS $p>0.05$ vs vehicle treatment (Repeated measures 2-way ANOVA + Bonferroni *post hoc* test).

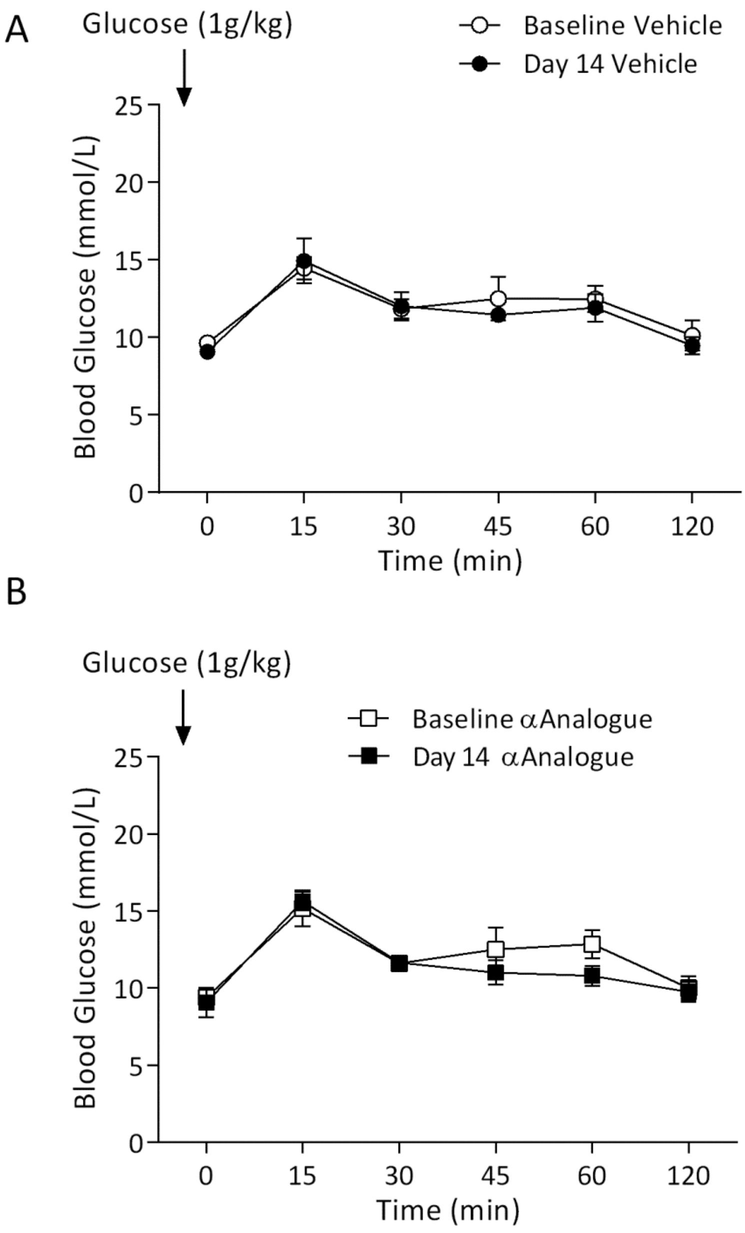

Supplemental Figure 9. Effects of cardiovascular active dose of α-CGRP analogue (αAnalogue) on glucose tolerance test in mice. Time course of plasma glucose concentrations (mmol/L) following systemic administration of glucose (1g/kg, *i.p.*) in mice treated daily with **(A)** vehicle (n=4) and **(B)** αAnalogue (50nmol/kg/day, *s.c.*, n=5) for 14 days. Results show mean ± SEM. Arrow denotes treatment time of glucose administration. NS p>0.05 vs baseline (Repeated measures 2-way ANOVA + Bonferroni post *hoc* test).

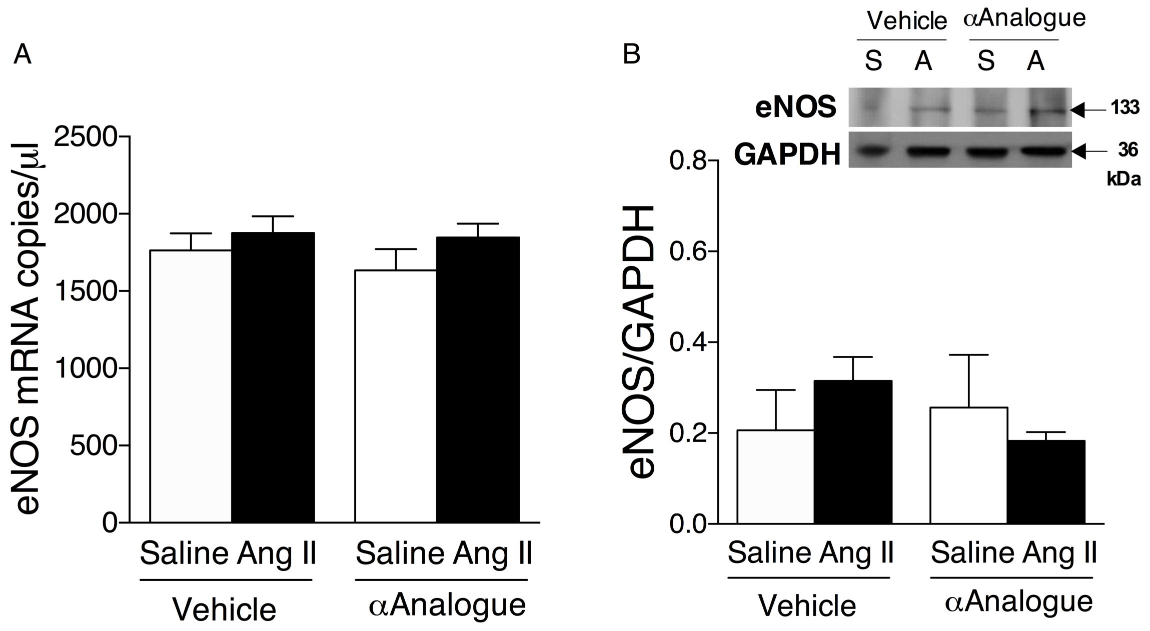

Supplemental Figure 10. Effects of α-CGRP analogue (αAnalogue) on vascular eNOS expression in mesenteric vessels. Mice were treated daily with the α-CGRP analogue (50nmol/kg, *s.c.*) or vehicle post AngII or saline infusion for 14 days. mRNA expression measured by qRT-PCR (n=4-6) and expressed as copy numbers per µl normalised to HPRT, B_2M and β-actin. Protein expression normalised by GAPDH and shown by immunoblotting (top panel) and densitometry analysis (bottom panel). (**A**) mRNA and **(B)** protein expression of endothelial nitric oxide synthase (eNOS). Results show mean $\pm$ SEM. ns $p>0.05$ (2-way ANOVA + Bonferroni *post hoc* test).

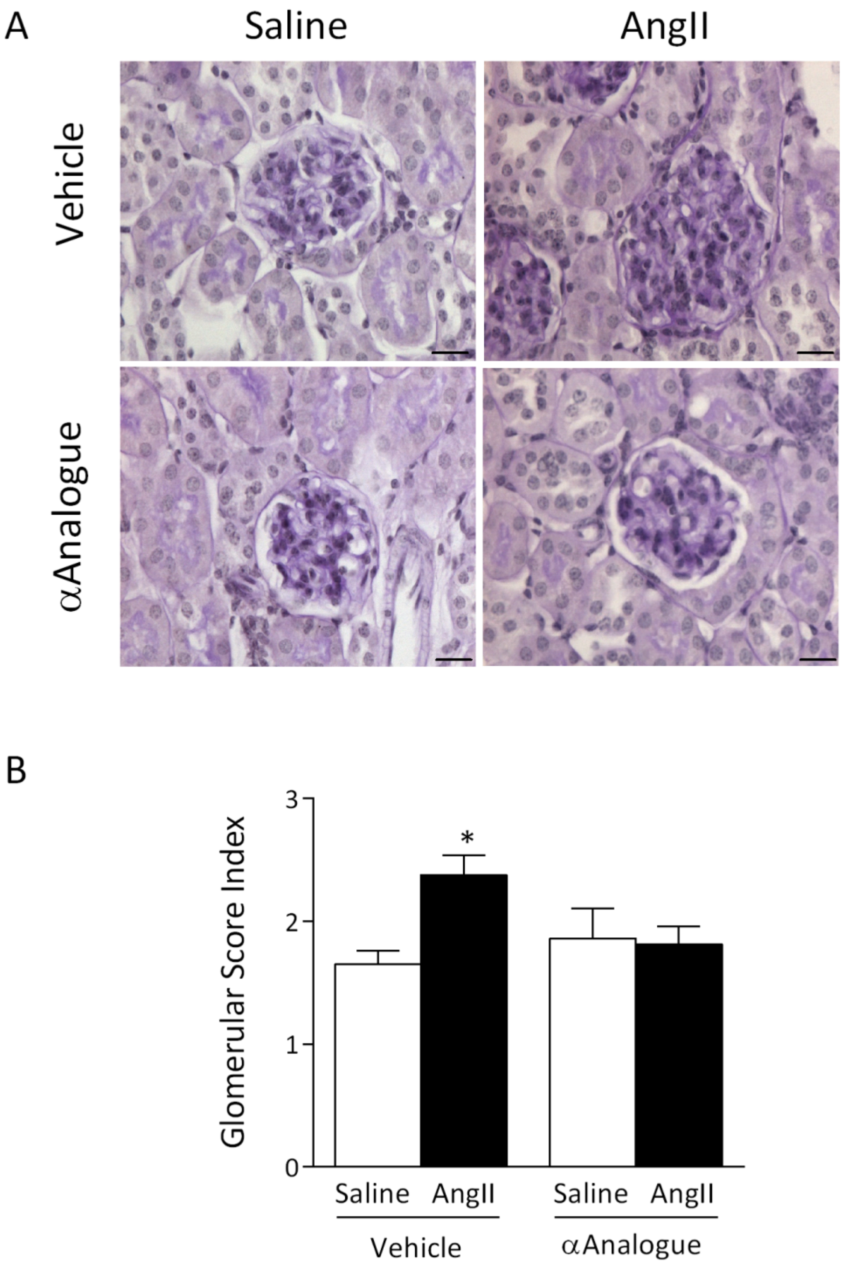

Supplemental Figure 11. Effects of α-CGRP analogue (αAnalogue) on morphological changes in the glomeruli in the kidney of Angiotensin II (AngII)-induced hypertension mice. Mice were treated daily with the αAnalogue (50nmol/kg, *s.c.*) or vehicle post AngII or saline infusion for 14 days. **(A)** Representative image showing glomerular mesangial pathology using PAS staining (20µm, scale bar). **(B)** Summarized glomerular matrix expansion by semi-quantification of scores in different groups. Results show mean $\pm$ SEM. *$p<0.05$ vs vehicle-treated (2-way ANOVA + Bonferroni *post hoc* test).

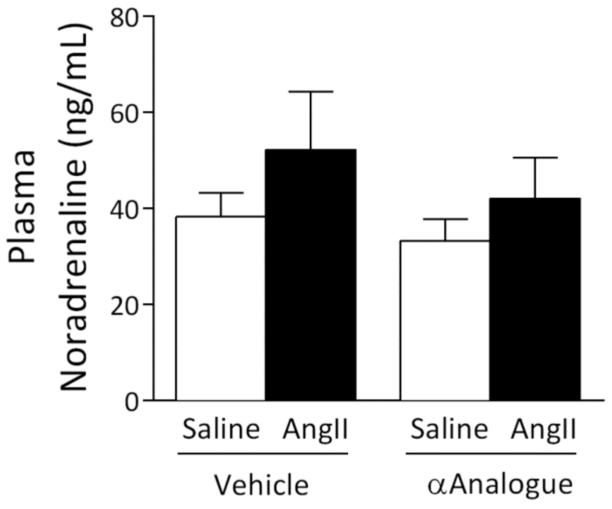

Supplemental Figure 12. Effects of α-CGRP analogue (αAnalogue) on plasma noradrenaline (NA) level in Angiotensin II(AngII)-induced hypertension mice. Mice were treated daily with the αAnalogue (50nmol/kg, *s.c.*) or vehicle post AngII or saline infusion for 14 days. NA was quantified using ELISA (n=6-9). Results show mean $\pm$ SEM. ns $p>0.05$ (2-way ANOVA + Bonferroni *post hoc* test).

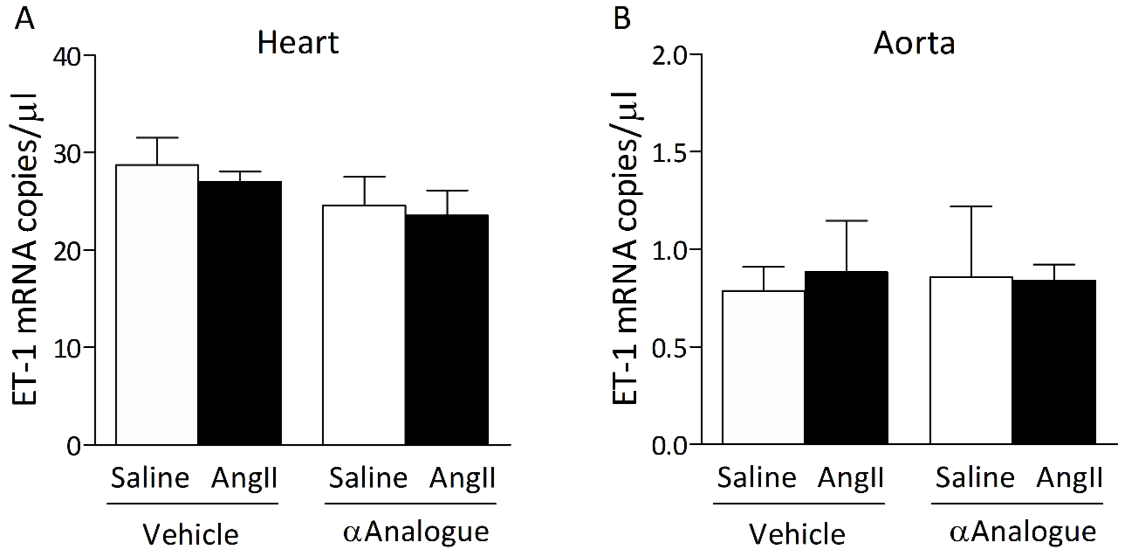

Supplemental Figure 13. Effects of α-CGRP analogue (αAnalogue) on mRNA expression of endothelial-1 (ET-1) level in Angiotensin II(AngII)-induced hypertension mice. Mice were treated daily with the αAnalogue (50nmol/kg, *s.c.*) or vehicle post AngII or saline infusion for 14 days. mRNA expression measured by qRT-PCR (n=6-7) for ET-1 in **(A)** heart and **(B)** aorta (n=4-11). Results expressed as copy numbers per µl normalised to HPRT, B2M and β-actin. Results show mean $\pm$ SEM. ns $p>0.05$ (2-way ANOVA + Bonferroni *post hoc* test).

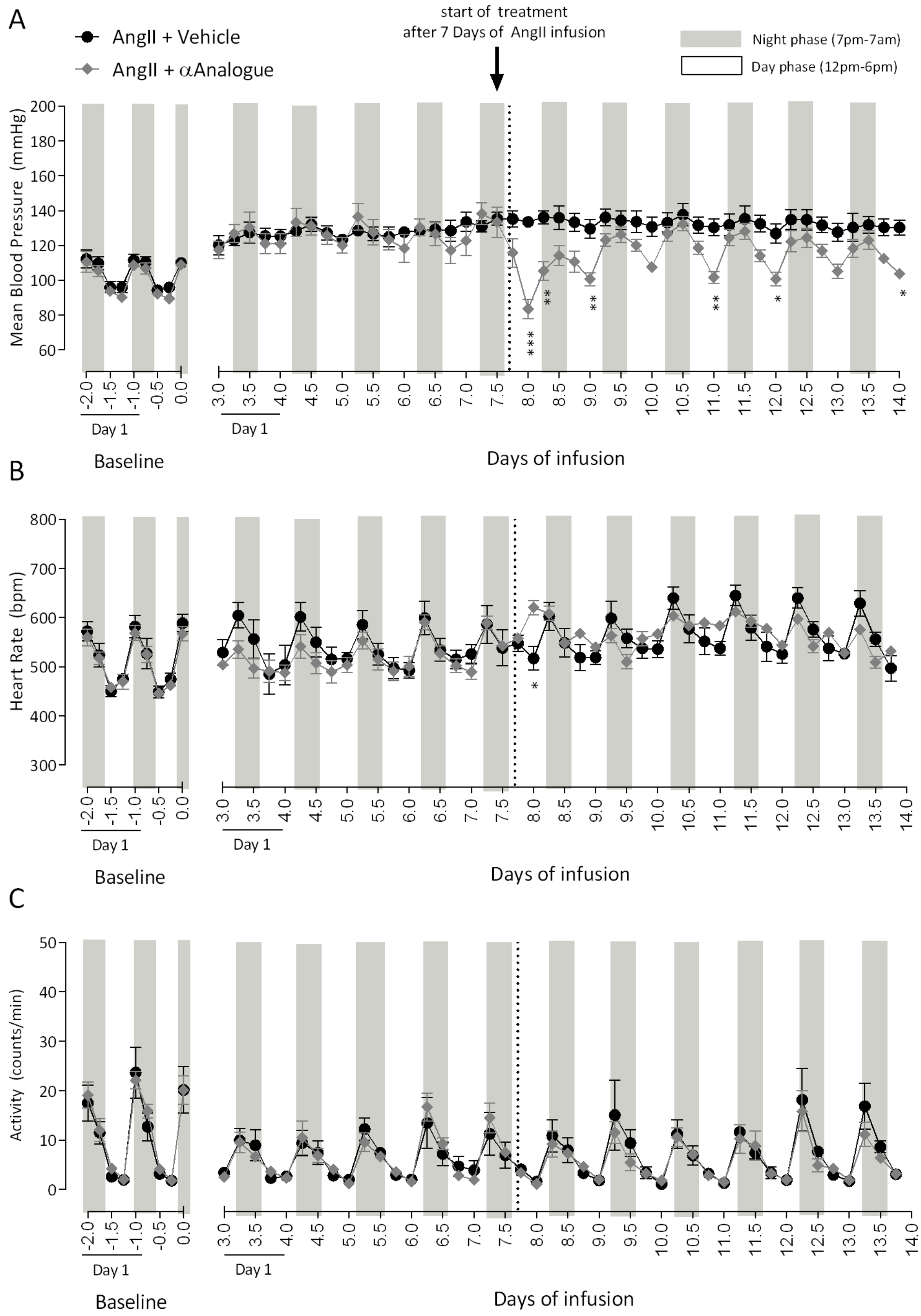

Supplemental Figure 14. Effect of daily treatment with α-CGRP analogue (αAnalogue, 50nmol/kg) on angiotensin II (AngII)-induced hypertensive mice. Mice were infused with AngII (1.1mg/kg/day) osmotic pumps for 14 days and treated with vehicle or αAnalogue (50nmol/kg) on Day 7-14 (n=4). **(A)** Mean blood pressure, **(B)** heart rate and **(C)** activity were measured by radiotelemetry. Results show measurement taken every 10 min, expressed as 6h average. Mice experience a 12/12h light/dark cycle, with the dark cycle shown in the grey striped area. Arrow indicates the start of daily treatment with vehicle or αAnalogue. *$p<0.05$, **$p<0.01$, ***$p<0.001$ vs vehicle-treated AngII-infused mice (Repeated measures 2-way ANOVA + Bonferroni *post hoc* test).

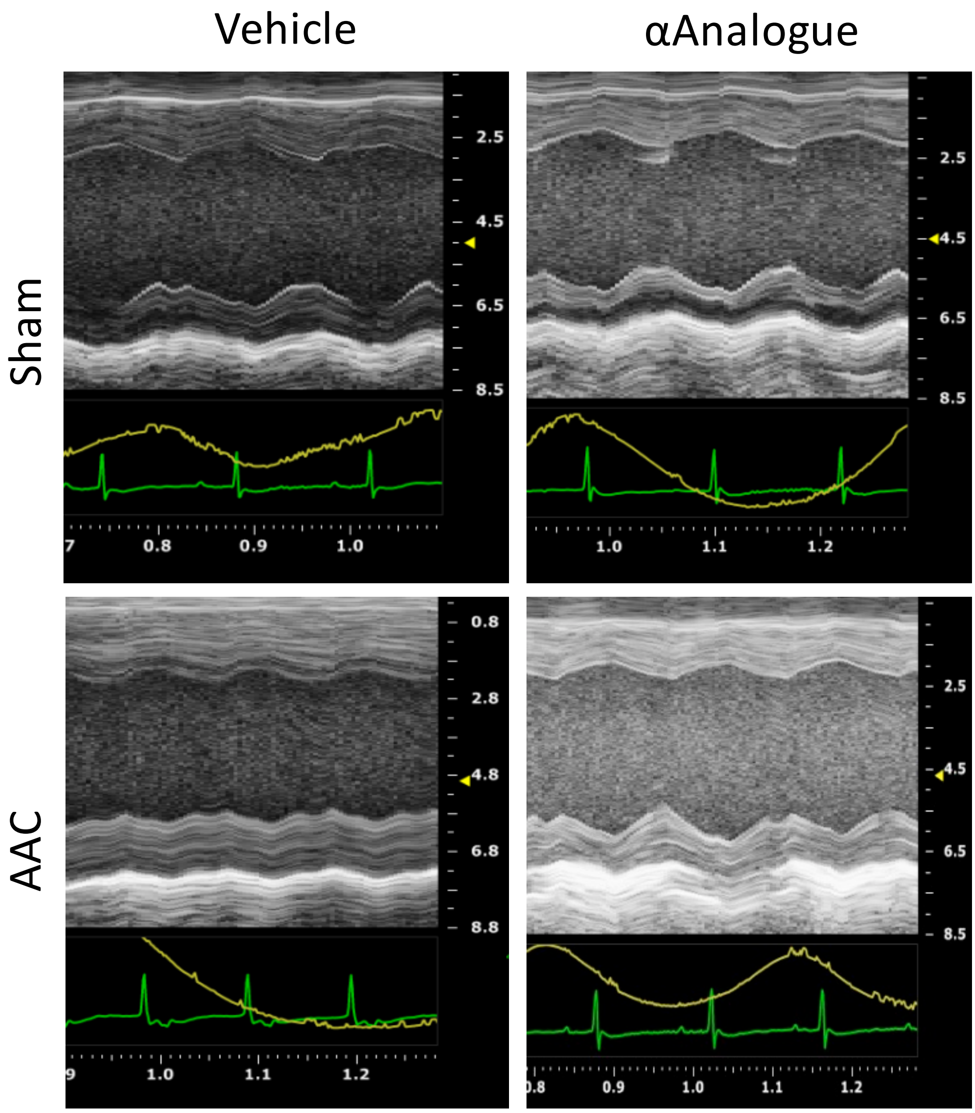

Supplemental Figure 15. Effect of daily treatment with α-CGRP analogue (αAnalogue) on abdominal aortic constriction (**AAC)-induced cardiac hypertrophy and heart failure in mice.** Mice were treated with vehicle or αAnalogue (50nmol/kg, *s.c.*) for 5 weeks post-surgery and cardiac function was assessed using echocardiography at week 5. Representative images of cardiac function and dimension using M-Mode echocardiography in the parasternal long axis view in mice after sham or AAC surgery with or without treatment.

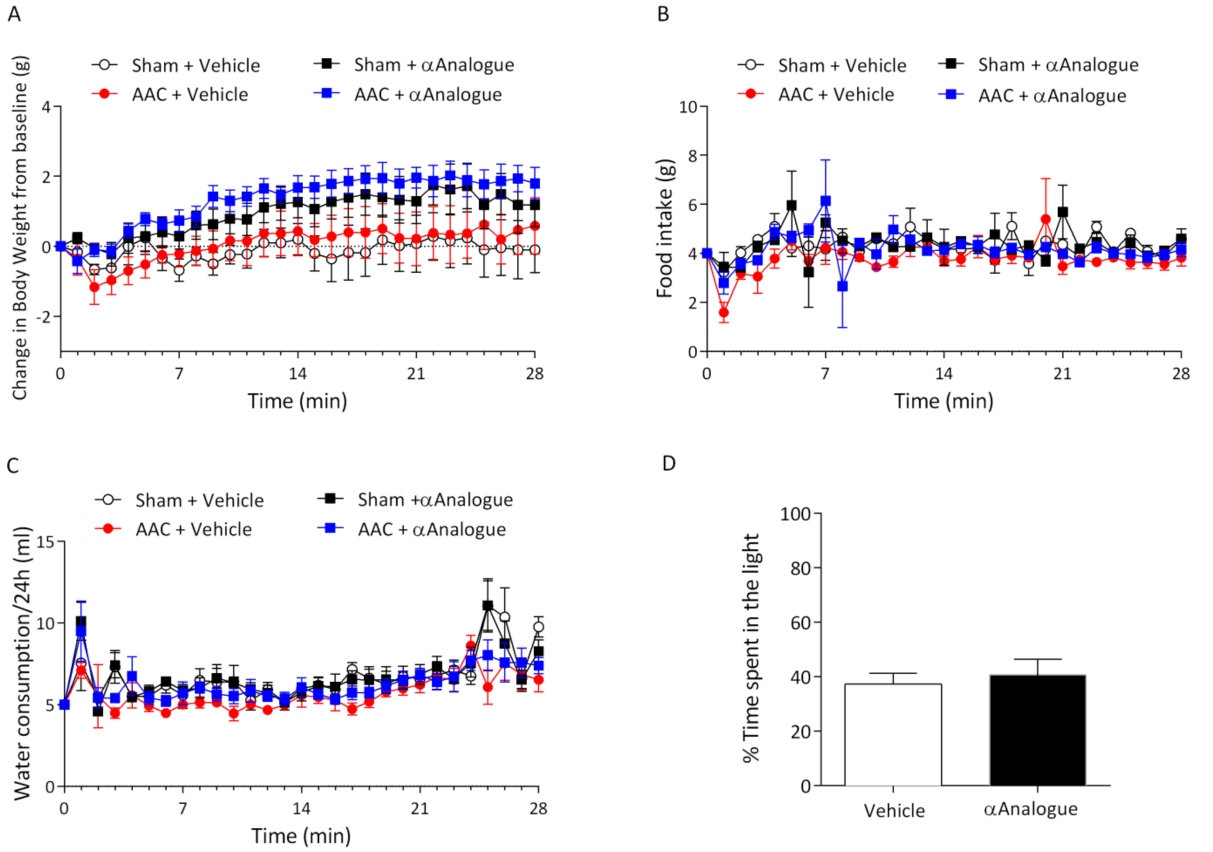

Supplemental Figure 16. Effects of the α-CGRP analogue (αAnalogue) or vehicle on (A) body weight, (B) food intake, (C) water consumption and (D) light aversion assay in abdominal aortic constriction-induced cardiac hypertrophy and heart failure. Mice were treated with vehicle or αAnalogue (50nmol/kg, *s.c.*) for 5 weeks post-surgery. Measurements were taken daily. Data are mean ± S.E.M from n=6-8. ns $p>0.05$ (Repeated measures 2-way ANOVA + Bonferroni *post hoc* test).

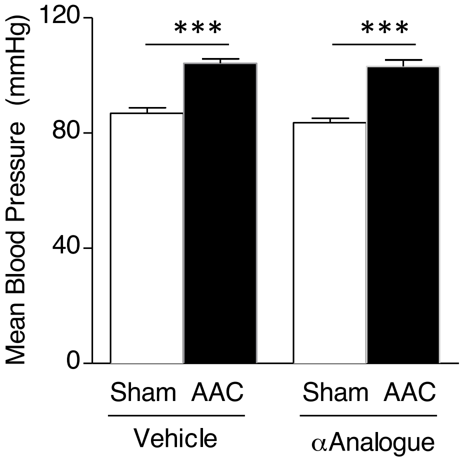

Supplemental Figure 17. Changes in blood pressure in vehicle or α-CGRP analogue (αAnalogue)-treated at 5 weeks following abdominal aorta constriction-induced cardiac hypertrophy and heart failure in mice. Blood pressure measurement obtained by carotid artery cannulation in isoflurane-anaesthetised mice treated daily with the αAnalogue (50nmol/kg, *s.c.*) or vehicle for 5 weeks (n=7-8). Results show mean $\pm$ SEM. ***p<0.001 vs vehicle treated sham mice (2-way ANOVA + Bonferroni *post hoc* test).

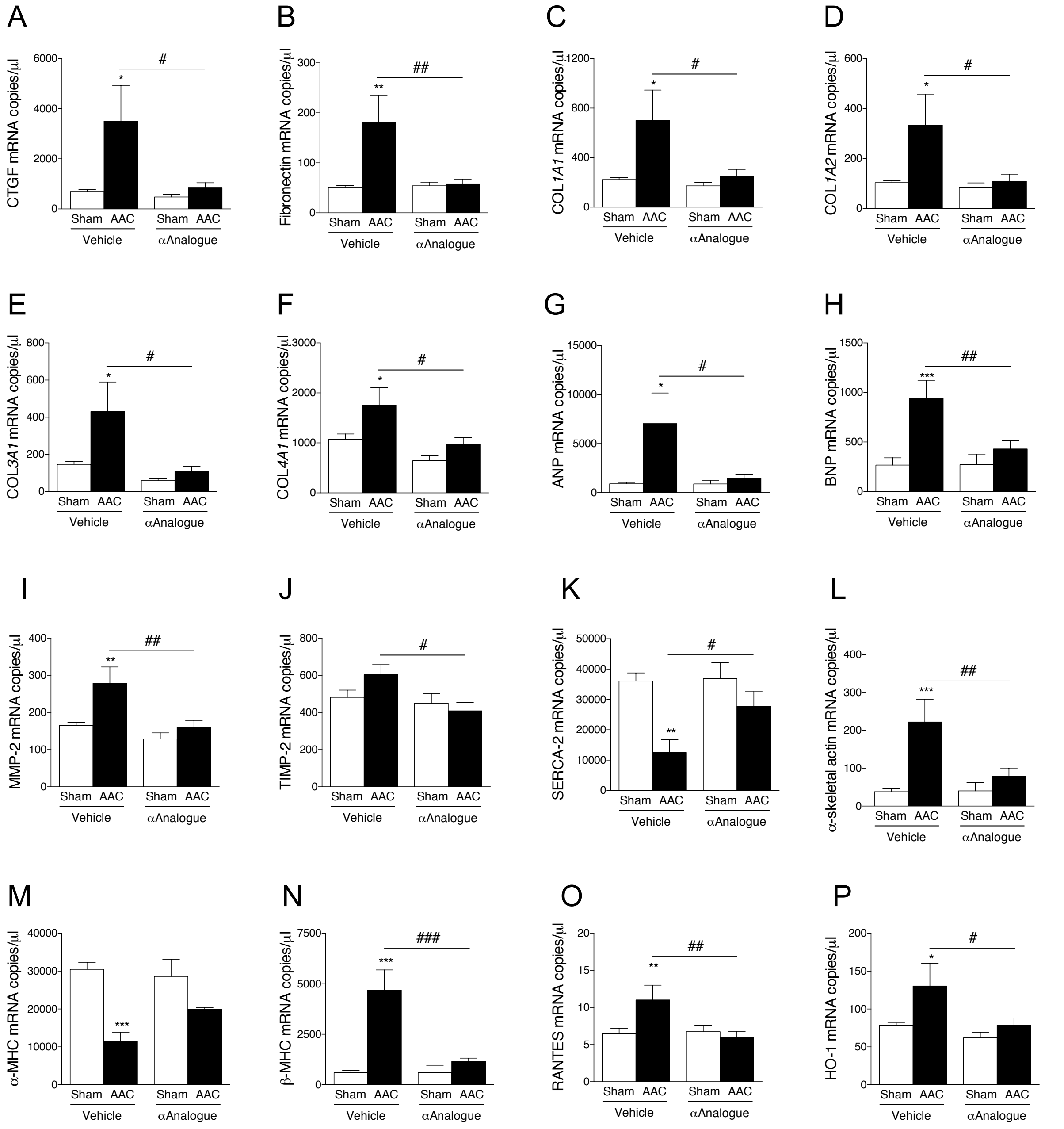

Supplemental Figure 18. Daily systemic treatment with α-CGRP analogue (αAnalogue) protects against abdominal aorta constriction (AAC)-induced cardiac fibrosis, hypertrophy, inflammation and oxidative stress. Following AAC, mice were treated daily for 5 weeks with vehicle or αAnalogue (50nmol/kg, *s.c.*). mRNA expression measured by qRT-PCR (n=6-7) for **(A)** CTGF, **(B)** fibronectin, **(C)** collagen type 1 α (COL*1A1*), **(D)** collagen type 1 α2 (COL*1A2*), **(E)** collagen type 3 α1 (COL*3A1*), **(F)** collagen type 4 α1 (COL*4A1*), **(G)** atrial natriuretic peptide (ANP), **(H)** brain natriuretic peptide (BNP), **(I)** matrix metalloproteinase-2 (MMP-2), **(J)** tissue inhibitor of metalloproteinase 2 (TIMP-2), **(K)** sarco-endoplasmic reticulum Ca^{2+} ATPase-2 (SERCA-2), **(L)** α-skeletal actin, **(M)** α-myosin heavy chain (α-MHC), **(N)** β-myosin heavy chain (β-MHC), **(O)** chemokine RANTES and **(P)** heme oxygenase-1 (HO-1) in heart (n=6-7). Results expressed as copy numbers per µl normalised to HPRT, B2M and β-actin. Results show mean ± SEM. *p<0.05, **p<0.01, ***p<0.001 vs vehicle-treated sham mice; #p<0.05, ##p<0.01, ###p<0.001 vs vehicle-treated AAC mice (2-WAY ANOVA + Bonferroni *post hoc* test).

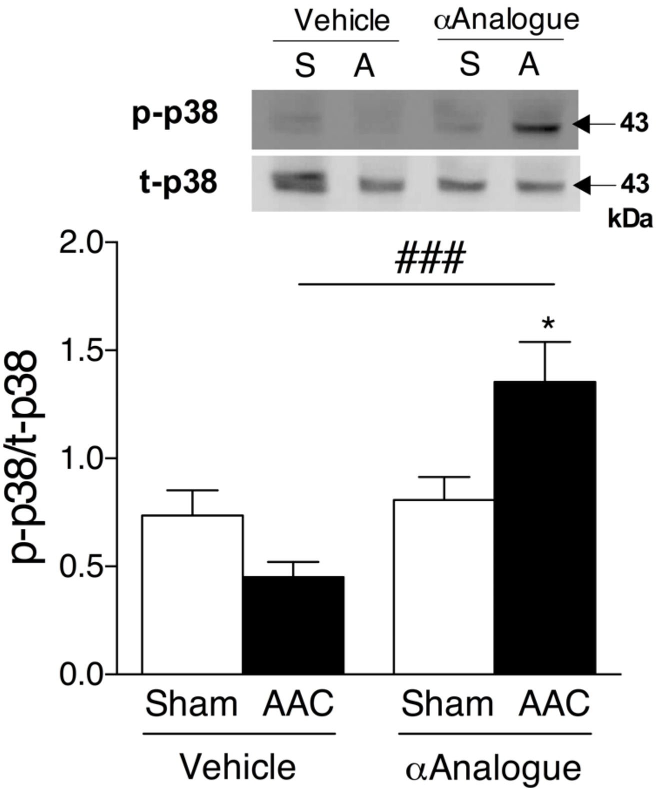

Supplemental Figure 19. Effects of α-CGRP analogue (αAnalogue) on the phosphorylation of p-38 mitogen-activated protein kinases (MAPK) in the heart of abdominal aortic constriction-induced cardiac hypertrophy and hear failure in mice. Mice were treated daily with the αAnalogue (50nmol/kg, *s.c.*) or vehicle post-surgery. Protein expression of phosphorylated p-38 was normalised to total p-38 (t-p38) and are shown by immunoblotting (top panel) and densitometry analysis (bottom panel) in heart tissues (n=6-8). Results show mean ± mean. *p<0.05 vs sham-treated, ###p<0.001 vs sham-treated (2-WAY ANOVA + Bonferroni *post hoc* test).

Supplemental References

1. Aubdool AA, Graepel R, Kodji X, Alawi KM, Bodkin JV, Srivastava S, Gentry C, Heads R, Grant AD, Fernandes ES, Bevan S and Brain SD. TRPA1 is essential for the vascular response to environmental cold exposure. *Nat Commun*. 2014;5:5732. doi:10.1038/ncomms6732

2. Aubdool AA, Kodji X, Abdul-Kader N, Heads R, Fernandes ES, Bevan S and Brain SD. TRPA1 activation leads to neurogenic vasodilatation: Involvement of reactive oxygen nitrogen species in addition to CGRP and NO. *Br J Pharmacol*. 2016;173:2419-2433. doi:10.1111/bph.13519

3. Alawi KM, Russell FA, Aubdool AA, Srivastava S, Riffo-Vasquez Y, Baldissera L, Jr., Thakore P, Saleque N, Fernandes ES, Walsh DA and Brain SD. Transient receptor potential canonical 5 (TRPC5) protects against pain and vascular inflammation in arthritis and joint inflammation. *Ann Rheum Dis*. 2016;76:252-260. doi:10.1136/annrheumdis-2015-208886

4. Doods H, Hallermayer G, Wu D, Entzeroth M, Rudolf K, Engel W and Eberlein W. Pharmacological profile of BIBN4096BS, the first selective small molecule CGRP antagonist. *Br J Pharmacol*. 2000;129:420-423. doi:10.1038/sj.bjp.0703110

5. Starr A, Graepel R, Keeble J, Schmidhuber S, Clark N, Grant A, Shah AM and Brain SD. A reactive oxygen species-mediated component in neurogenic vasodilatation. *Cardiovasc Res*. 2008;78:139-147. doi:10.1093/cvr/cvn012

6. Nielsen AS, Kruse T, Kodra JT, Lau JF, Kofoed J, Raun K and Nilsson C, inventors; Novo Nordisk, assignee. Derivatives of cgrp. WO patent 2,011,051,312. May 5, 2011.

7. Smillie SJ, King R, Kodji X, Outzen E, Pozsgai G, Fernandes E, Marshall N, de Winter P, Heads RJ, Dessapt-Baradez C, Gnudi L, Sams A, Shah AM, Siow RC and Brain SD. An ongoing role of alpha-calcitonin gene-related peptide as part of a protective network against hypertension, vascular hypertrophy, and oxidative stress. *Hypertension*. 2014;63:1056-1062. doi:10.1161/HYPERTENSIONAHA.113.02517

8. Marshall NJ, Liang L, Bodkin J, Dessapt-Baradez C, Nandi M, Collot-Teixeira S, Smillie SJ, Lalgi K, Fernandes ES, Gnudi L and Brain SD. A role for TRPV1 in influencing the onset of cardiovascular disease in obesity. *Hypertension*. 2013;61:246-252. doi:10.1161/HYPERTENSIONAHA.112.201434

9. Leiper J, Nandi M, Torondel B, Murray-Rust J, Malaki M, O'Hara B, Rossiter S, Anthony S, Madhani M, Selwood D, Smith C, Wojciak-Stothard B, Rudiger A, Stidwill R, McDonald NQ and Vallance P. Disruption of methylarginine metabolism impairs vascular homeostasis. *Nat Med*. 2007;13:198-203. doi:10.1038/nm1543

10. Bodkin JV, Thakore P, Aubdool AA, Liang L, Fernandes ES, Nandi M, Spina D, Clark JE, Aaronson PI, Shattock MJ and Brain SD. Investigating the potential role of TRPA1 in locomotion and cardiovascular control during hypertension. *Pharmacol Res Perspect*. 2014;2:e00052. doi:10.1002/prp2.52

11. Zhang M, Brewer AC, Schroder K, Santos CX, Grieve DJ, Wang M, Anilkumar N, Yu B, Dong X, Walker SJ, Brandes RP and Shah AM. NADPH oxidase-4 mediates protection against chronic load-induced stress in mouse hearts by enhancing angiogenesis. *Proc Natl Acad Sci U S A*. 2010;107:18121-18126. doi:10.1073/pnas.1009700107

12. Murray TV, Smyrnias I, Schnelle M, Mistry RK, Zhang M, Beretta M, Martin D, Anilkumar N, de Silva SM, Shah AM and Brewer AC. Redox regulation of cardiomyocyte cell cycling via an ERK1/2 and c-Myc-dependent activation of cyclin D2 transcription. *J Mol Cell Cardiol*. 2015;79:54-68. doi:10.1016/j.yjmcc.2014.10.017

13. Crawley J and Goodwin FK. Preliminary report of a simple animal behavior model for the anxiolytic effects of benzodiazepines. *Pharmacol Biochem Behav*. 1980;13:167-170.

14. Thiels E, Hoffman EK and Gorin MB. A reliable behavioral assay for the assessment of sustained photophobia in mice. *Curr Eye Res*. 2008;33:483-491. doi:10.1080/02713680802130347

15. Kaiser EA, Kuburas A, Recober A and Russo AF. Modulation of CGRP-induced light aversion in wild-type mice by a 5-HT(1B/D) agonist. *J Neurosci*. 2012;32:15439-15449. doi:10.1523/JNEUROSCI.3265-12.2012

16. Ramachandran R, Bhatt DK, Ploug KB, Olesen J, Jansen-Olesen I, Hay-Schmidt A and Gupta S. A naturalistic glyceryl trinitrate infusion migraine model in the rat. *Cephalalgia*. 2012;32:73-84. doi:10.1177/0333102411430855

17. Papacleovoulou G, Abu-Hayyeh S, Nikolopoulou E, Briz O, Owen BM, Nikolova V, Ovadia C, Huang X, Vaarasmaki M, Baumann M, Jansen E, Albrecht C, Jarvelin MR, Marin JJ, Knisely AS and Williamson C. Maternal cholestasis during pregnancy programs metabolic disease in offspring. *J Clin Invest*. 2013;123:3172-3181. doi:10.1172/JCI68927

18. Alawi KM, Aubdool AA, Liang L, Wilde E, Vepa A, Psefteli MP, Brain SD and Keeble JE. The sympathetic nervous system is controlled by transient receptor potential vanilloid 1 in the regulation of body temperature. *FASEB J*. 2015;29:4285-4298. doi:10.1096/fj.15-272526

19. Looi YH, Grieve DJ, Siva A, Walker SJ, Anilkumar N, Cave AC, Marber M, Monaghan MJ and Shah AM. Involvement of Nox2 NADPH oxidase in adverse cardiac remodeling after myocardial infarction. *Hypertension*. 2008;51:319-325. doi:10.1161/HYPERTENSIONAHA.107.101980

20. Murdoch CE, Chaubey S, Zeng L, Yu B, Ivetic A, Walker SJ, Vanhoutte D, Heymans S, Grieve DJ, Cave AC, Brewer AC, Zhang M and Shah AM. Endothelial NADPH oxidase-2 promotes interstitial cardiac fibrosis and diastolic dysfunction through proinflammatory effects and endothelial-mesenchymal transition. *J Am Coll Cardiol*. 2014;63:2734-2741. doi:10.1016/j.jacc.2014.02.572

21. Liang L, Tam CW, Pozsgai G, Siow R, Clark N, Keeble J, Husmann K, Born W, Fischer JA, Poston R, Shah A and Brain SD. Protection of angiotensin II-induced vascular hypertrophy in vascular smooth muscle-targeted receptor activity-modifying protein 2 transgenic mice. *Hypertension*. 2009;54:1254-1261. doi:10.1161/HYPERTENSIONAHA.109.129783

22. Junqueira LC, Bignolas G and Brentani RR. Picrosirius staining plus polarization microscopy, a specific method for collagen detection in tissue sections. *Histochem J*. 1979;11:447-455.

23. Ducharme A, Frantz S, Aikawa M, Rabkin E, Lindsey M, Rohde LE, Schoen FJ, Kelly RA, Werb Z, Libby P and Lee RT. Targeted deletion of matrix metalloproteinase-9 attenuates left ventricular enlargement and collagen accumulation after experimental myocardial infarction. *J Clin Invest*. 2000;106:55-62. doi:10.1172/JCI8768

24. Riehle C, Wende AR, Zaha VG, Pires KM, Wayment B, Olsen C, Bugger H, Buchanan J, Wang X, Moreira AB, Doenst T, Medina-Gomez G, Litwin SE, Lelliott CJ, Vidal-Puig A and Abel ED. PGC-1beta deficiency accelerates the transition to heart failure in pressure overload hypertrophy. *Circ Res*. 2011;109:783-793. doi:10.1161/CIRCRESAHA.111.243964

25. Johnson RJ, Iida H, Alpers CE, Majesky MW, Schwartz SM, Pritzi P, Gordon K and Gown AM. Expression of smooth muscle cell phenotype by rat mesangial cells in immune complex nephritis. Alpha-smooth muscle actin is a marker of mesangial cell proliferation. *J Clin Invest*. 1991;87:847-858. doi:10.1172/JCI115089

26. Bank N, Klose R, Aynedjian HS, Nguyen D and Sablay LB. Evidence against increased glomerular pressure initiating diabetic nephropathy. *Kidney Int*. 1987;31:898-905.
